# Vitamin D in pediatric age: consensus of the Italian Pediatric Society and the Italian Society of Preventive and Social Pediatrics, jointly with the Italian Federation of Pediatricians

**DOI:** 10.1186/s13052-018-0488-7

**Published:** 2018-05-08

**Authors:** Giuseppe Saggese, Francesco Vierucci, Flavia Prodam, Fabio Cardinale, Irene Cetin, Elena Chiappini, Gian Luigi de’ Angelis, Maddalena Massari, Emanuele Miraglia Del Giudice, Michele Miraglia Del Giudice, Diego Peroni, Luigi Terracciano, Rino Agostiniani, Domenico Careddu, Daniele Giovanni Ghiglioni, Gianni Bona, Giuseppe Di Mauro, Giovanni Corsello

**Affiliations:** 1 Department of Clinical and Experimental Medicine, Section of Paediatrics, University of Pisa, Pisa, Italy; 2 Pediatric Unit, San Luca Hospital, Lucca, Italy; 3 Division of Pediatrics, Department of Health Sciences, Interdisciplinary Research Center of Autoimmune Diseases (IRCAD), University of Piemonte Orientale, Novara, Italy; 4 Pediatric Unit, Division of Pulmonology, Allergy, and Immunology, AOU Policlinico-Giovanni XXIII, Bari, Italy; 5 Department of Mother and Child, Hospital Luigi Sacco, University of Milano, Milan, Italy; 6 Pediatric Infectious Disease Unit, Department of Health Sciences, University of Florence, Anna Meyer Children’s University Hospital, Florence, Italy; 7 Gastroenterology and Digestive Endoscopy Unit and Clinical Paediatrics Unit, Department of Paediatrics and Maternal Medicine, University of Parma Hospital Trust, Parma, Italy; 8 Department of Woman, Child and General and Specialist Surgery, University of Campania “Luigi Vanvitelli”, Naples, Italy; 9 Pediatric Primary Care, National Pediatric Health Care System, Milan, Italy; 10 Department of Pediatrics, San Jacopo Hospital, Pistoia, Italy; 11 Pediatric Primary Care, National Pediatric Health Care System, Novara, Italy; 12 Pediatric Highly Intensive Care Unit, Department of Pathophysiology and Transplantation, Università degli Studi di Milano, Fondazione IRCCS Ca’ Granda Ospedale Maggiore Policlinico, Milan, Italy; 13 Division of Pediatrics, University of Piemonte Orientale, Novara, Italy; 14 Pediatric Primary Care, National Pediatric Health Care System, Caserta, Italy; 15 Department of Sciences for Health Promotion and Mother and Child Care, Neonatal Intensive Care Unit, AOUP, University of Palermo, Palermo, Italy

**Keywords:** Vitamin D, Supplementation, Children, Adolescents, Deficiency, Hypovitaminosis D

## Abstract

Vitamin D plays a pivotal role in the regulation of calcium-phosphorus metabolism, particularly during pediatric age when nutritional rickets and impaired bone mass acquisition may occur.

Besides its historical skeletal functions, in the last years it has been demonstrated that vitamin D directly or indirectly regulates up to 1250 genes, playing so-called extraskeletal actions. Indeed, recent data suggest a possible role of vitamin D in the pathogenesis of several pathological conditions, including infectious, allergic and autoimmune diseases. Thus, vitamin D deficiency may affect not only musculoskeletal health but also a potentially wide range of acute and chronic conditions. At present, the prevalence of vitamin D deficiency is high in Italian children and adolescents, and national recommendations on vitamin D supplementation during pediatric age are lacking. An expert panel of the Italian Society of Preventive and Social Pediatrics reviewed available literature focusing on randomized controlled trials of vitamin D supplementation to provide a practical approach to vitamin D supplementation for infants, children and adolescents.

## Background

Vitamin D plays a fundamental role in regulating calcium and phosphorus homeostasis and, in particular, the pathways involved in bone mineralization and bone mass acquisition. Besides these classic skeletal actions, recent studies have demonstrated that vitamin D exerts other significant extraskeletal actions, with a possible role in the pathogenesis of several pathological conditions, including infectious and autoimmune diseases [1]. The term ‘vitamin D’ is used for two different forms which are found in nature: vitamin D_3_ (cholecalciferol) from animal sources and vitamin D_2_ (ergocalciferol) from plants. Humans synthesize vitamin D_3_ in their skin in response to sunlight exposure and vitamin D_2_ and D_3_ may be supplied from dietary sources, although only a few foods contain significant amounts (Table 1). Thus, with the exclusion of artificially fortified foods, the contribution of dietary intakes may be considered negligible [2, 3]. Previtamin D_3_ is produced in the skin following ultraviolet B irradiation (at wavelengths between 290 and 315 nm) of the 7-dehydrocholesterol present in all the layers of human skin, mainly in the epidermis. Previtamin D_3_ is an unstable molecule, subsequently transformed in vitamin D_3_ by a process of thermo-conversion. Once previtamin D_3_ is synthesized in the skin, it can undergo also a photoconversion to lumisterol and tachysterol, solar photoproducts inactive on calcium metabolism that are produced at times of prolonged exposure to solar UV-B radiation, to prevent sun-induced vitamin D intoxication. Vitamin D_2_ and D_3_ are transported by vitamin D binding protein to the liver, where they are 25-hydroxylated by the vitamin D-25-hydroxylase (CYP2R1) to produce 25-hydroxyvitamin D [25(OH)D], the major circulating vitamin D metabolite which is used to evaluate individual vitamin D status. Then 25(OH)D reaches the kidney, where it undergoes a further hydroxylation by 25(OH)D-1α-hydroxylase (CYP27B1) into 1,25-dihydroxyvitamin D [1,25(OH)_2_D or calcitriol], the bioactive hormonal form of vitamin D [2, 4]. Calcitriol is able to regulate calcium-phosphorus balance in various pathways, first stimulating calcium and phosphorus absorption by enterocytes. When dietary calcium intakes are inadequate, calcitriol interacts with the vitamin D receptor (VDR) expressed on osteoblasts, bringing osteoclasts precursors to maturation and promoting calcium and phosphorus absorption by bone tissue. Calcitriol acts synergistically with parathormone (PTH) that acts in bone stimulating calcium absorption by the osteoclasts, and in the kidney where it promotes calcium reuptake in the tubules, phosphorus excretion, and vitamin D conversion into its active hormone form [2].

**Table 1:** Vitamin D content in some foods

Food	Vitamin D average content (IU)
Milk and dairy products	
Cow’s milk	5–40/l
Goat’s milk	5–40/l
Butter	30/100 g
Yogurt	2.4/100 g
Cream	30/100 g
Other Foods	
Pork	40–50/100 g
Beef liver	40–50/100 g
Snapper (genus *Dentex dentex*), cod, gilthead (*Sparus auratus*), dogfish (*Mustelus mustelus*), sole, trout, salmon, herring	300–1500/100 g
Cod liver oil	400/5 ml
Egg yolk	20/100 g

## Methods

This document represents a consensus opinion of experts derived from current literature revision and it is intended to be mainly directed to hospital or primary care pediatricians. The main objective is to give recommendations regarding the prevention and treatment of vitamin D deficiency in Italian children and adolescents (0–18 years), considering both the skeletal and extra-skeletal effects of vitamin D and potential risk factors in specific subgroups of children. The Working Group agreed on a list of relevant clinical topics. Using the Consensus Conference method based on the National Institutes of Health and the Italian National Programme Guidelines [5, 6], relevant publications in English limited to the pediatric age (< 18 years) were identified by a review of MEDLINE by PubMed, from 1st January 2005 to 1st August 2017, and using appropriate search strategies for each topic. Checklists and predefined tables were used to assess study quality and to extract data in a standard way. Further literature search was performed focusing available international guidelines on vitamin D supplementation in children. The panel will be taking up the issue again in 2 years, and will promote a new consensus conference if clinically relevant evidence has emerged from new studies. The full text of the guidelines and related documents in Italian are available at the site of the Italian Society of Preventive and Social Pediatrics [7].

## Vitamin D deficiency: Ranges, analytic methods, and epidemiology

The major circulating form of vitamin D is 25(OH)D, having a half-life of 2–3 weeks. It is the best marker to monitor for vitamin D status. In literature, several cut-offs have been proposed for vitamin D deficiency with respect to serum 25(OH)D levels measured with a reliable assay. These cut-offs derive from PTH feedback threshold, calcium intestinal absorption and bone health (presence of rickets/osteomalacia, low bone mass and/or mineral content, fracture risk) [8–30] (Table 2). On the other hand, the majority of the studies have been realized in adults.

**Table 2:** Definition of vitamin D status according to several Societies and Organizations in the last 10 years

Society/Organization	Year	Severe deficiency	Deficiency	Insufficiency	Sufficiency/Adequacy
Canadian Pediatric Society [8]	2007	–	< 10 ng/ml	10–29 ng/ml	≥ 30 ng/ml
Lawson Wilkins Pediatric Endocrine Society [9]	2008	< 5 ng/ml	5–14 ng/ml	15–19 ng/ml	≥ 20 ng/ml
Institute of Medicine [10]	2011	–	< 12 ng/ml	12–20 ng/ml^a^	≥ 20 ng/ml
The Endocrine Society [11]	2011	–	< 20 ng/ml	21–29 ng/ml	≥ 30 ng/ml
British Paediatric and Adolescent Bone Group [12]	2012	–	< 10 ng/ml	10–19 ng/ml	≥ 20 ng/ml
French Society of Paediatrics [13]	2012	–	< 20 ng/ml	–	≥ 20 ng/ml
Asociación Espanola de Pediatría (Spain) [14]	2012	–	< 20 ng/ml	–	≥ 20 ng/ml
Federal Commission for Nutrition (Switzerland) [15]	2012	< 10 ng/ml	< 20 ng/ml	–	≥ 20 ng/ml
Nordic Nutrition Recommendations [16]	2012	–	< 12 ng/ml	12–20 ng/ml	≥ 20 ng/ml
German Nutrition Society [17]	2012	–	–	–	≥ 20 ng/ml
Health council of the Netherlands [18]	2012	–	–	–	≥ 12 ng/ml
European Society for Paediatric Gastroenterology Hepatology and Nutrition [19]	2013	< 10 ng/ml	< 20 ng/ml	–	≥ 20 ng/ml
Central Europe [20]	2013	–	< 20 ng/ml	20–29 ng/ml	≥ 30 ng/ml
Society for Adolescent Health and Medicine [21]	2013	–	< 20 ng/ml	20–29 ng/ml	≥ 30 ng/ml
Australia/New Zealand [22]	2013	< 5 ng/ml	5–11 ng/ml	12–19 ng/ml	≥ 20 ng/ml
American Academy of Pediatrics [23]	2014	–	< 20 ng/ml	–	≥ 20 ng/ml
Japanese Society for Bone and Mineral Research, Japan Endocrine Society^b^ [24]	2015	–	< 20 ng/ml	–	–
Scientific Advisory Committee on Nutrition [25]	2016	–	–	–	≥ 10 ng/ml
European Food Safety Authority [26]	2016	–	–	–	≥ 20 ng/ml
United Arab Emirates [27]	2016	–	< 20 ng/ml	20–29 ng/ml	≥ 30 ng/ml
Global Consensus for rickets [28]	2016	–	< 12 ng/ml	12–19 ng/ml	≥ 20 ng/ml
Japanese Society for Bone and Mineral Research, Japan Endocrine Society^c^ [29]	2017	–	< 20 ng/ml	20–29 ng/ml	≥ 30 ng/ml
European Academy of Pediatrics [30]	2017	Definition of vitamin D status is unclear due to a lack of consensus			

^a^vitamin D inadequacy

^b^Diagnostic criteria for rickets

^c^Assessment criteria for vitamin D deficiency/insufficiency (authors reported that different criteria may be needed for children)

Severe vitamin D deficiency is defined as 25(OH)D levels < 10 ng/ml by several Authors and Societies because the risk of rickets is high below this cut-off also in presence of an adequate calcium intake [31–33] (see chapter “Nutritional rickets”). The prevalence of rickets is also significant with 25(OH)D levels of 10–15 ng/ml in case of a low calcium intake [31, 33]. In the majority of studies in adults, 25(OH)D levels of at least 20 ng/ml meet the needs of at least 97.5% of the population with regards to bone health [10, 11], while serum 25(OH)D levels ≥30 ng/ml are sufficient in about 100% of the adults [10, 11]. Secondary hyperparathyroidism generally develops with 25(OH)D levels < 20 ng/ml, and PTH levels reach a plateau at 25(OH)D levels of 30–40 ng/ml [34–37]. However, some Authors showed that the relationship between PTH and 25(OH)D levels may be influenced by many factors as age, gender, ethnicity, and weight [38, 39]. Few studies evaluated the relationship between 25(OH)D and PTH in pediatrics, thus defining a threshold is even more complex [40–48]. A study on 1370 Canadian infants (1–6 years) observed the PTH plateau at 25(OH)D levels > 42.8 ng/ml [49], whereas an Italian study on children and adolescents reported a lower prevalence of secondary hyperparathyroidism at 25(OH)D levels of 20–29 ng/ml, but no cases were detected above 30 ng/ml [50]. Moreover, no clear evidence of a threshold for calcium absorption was found with several 25(OH)D ranges in pediatrics. This could be due by an age-dependent more efficient calcium absorption, a compensatory absorption vitamin D-independent and a higher conversion to calcitriol [44, 51, 52]. Also when bone health is considered, the studies are relatively small (see chapter “Bone health”). A positive association between 25(OH)D levels, bone mineral content (BMC), and bone mineral density (BMD) has been reported in children and adolescents, in particular from the peripuberty, without a specific threshold [53]. Moreover, a recent study in Chinese infants (0–7 years) showed that when serum 25(OH)D levels were above 30 ng/ml, the prevalence of low tibial BMD (assessed by quantitative ultrasound) reached a plateau [54]. Based on the above considerations, we suggest to define vitamin D status as reported in Table 3.

**Table 3:** Cut-off points for the definition of vitamin D status based on circulating levels of 25(OH)D

	Severe deficiency	Deficiency	Insufficiency	Sufficiency
25(OH)D	< 10 ng/ml (< 25 nmol/l)	< 20 ng/ml (< 50 nmol/l)	20–29 ng/ml (50–74 nmol/l)	≥ 30 ng/ml (≥ 75 nmol/l)

Conversion factor: ng/ml = nmol/l*0.401; nmol/l = ng/ml*2.496

Vitamin D status is defined by the measurement of 25(OH)D concentrations. This term refers to both its circulating forms, the 25(OH)D_3_ and 25(OH)D_2_, the last from plant dietary sources. 1,25(OH)_2_D measurement does not reflect vitamin D status, owing to the short half-life (4–6 h) and the lower concentration (pg/ml vs. ng/ml). 1,25(OH)_2_D levels are reduced only when 25(OH)D levels are below 4 ng/ml. The measurement of 25(OH)D is difficult due to its lipophilic nature, the binding to vitamin D binding protein, the different circulating forms that also include epimers and isobars, and the standardization. In particular, the 24,25-dihydroxyvitamin D may represent up to 10–15% of the total quantity of 25(OH)D [55]. Various methods are available for determining 25(OH)D concentration, from the low-throughput radioimmunoassay techniques to the new automated immunoassays with high capacity, which in some cases still present poor accuracy and precision [56, 57].The National Institute of Standards and Technology has developed the stock standards for the measurements of 25(OH)D_3_/D_2_ levels through isotope-dilution liquid chromatography-tandem mass spectrometry (LC-MS/MS) methods [58]. The issue of international standardization of serum 25(OH)D measurement is also being progressed by the Vitamin D Standardization Program. The standardization, also retrospectively, should help to develop future vitamin D guidelines [59, 60]. The isotope-dilution LC-MS/MS is the standard method to always use in the neonates, because it is able to detect the serum C3 epimer of 25(OH)D_3_, that may represent up to the 40% of the total quantity of 25(OH)D [61–63]. When a patient is under treatment with ergocalciferol, measurements able to detect 25(OH)D_2_ are needed to avoid the risk of hypervitaminosis D [64]. Because LC-MS/MS methods are not so frequently used in clinical practice, at the moment other reliable assays could be used and certified laboratories are suggested. The measurement of free 25(OH)D has not been standardized. Moreover, current algorithms to calculate free 25(OH)D may not be accurate [65].

Hypovitaminosis D and vitamin D deficiency, independently from cut-off definitions, have a higher prevalence worldwide in any age. In pediatrics, US data derived by the National Health and Nutrition Examination Survey cohort indicate a prevalence ranging 9–18%, and 51–61% of vitamin D deficiency and hypovitaminosis D, respectively [66, 67]. A recent meta-analysis was conducted on all the cohort studies of the European population, basing also on a pediatric population of 14971 subjects (1–18 years) [68]. The Authors applied the Vitamin D standardization Program and developed protocols for standardizing existing 25(OH)D values from national health/nutrition surveys. The prevalence according to age (1–6 years, 7–14 years, and 15–18 years) ranged 4–7%, 1–8%, and 12–40%, respectively, suggesting that particular attention should be kept not only in infants but also in adolescents. Non-white subjects and those living at relatively mild-latitude countries (47–60° N) had a higher prevalence range (5–20%) than southern countries. Limitations of the study include the fact that some of the studies mainly included children aged 7–11 years, and that vitamin D supplements, food fortification or sun awareness campaigns could have influenced the estimates. Data from Italian pediatrics are only limited being represented by the Roma cohort (12.5–17.5 years) included in the HELENA study [68].

Other Italian data are present in literature, although they show some limitations, regarding small populations, analytic methods for 25(OH)D, the season of recruitment, the prevalence of overweight/obesity, vitamin D supplements, sunscreens, ethnicity, and uncovered areas of latitude. Despite these points, Italian data are in line with those reported above, with higher prevalence in neonates, in particular in those not Caucasian, in adolescents and in overweight/obese subjects [50, 69–84]. Interestingly, also the more limited estimates in southern Italy parallel those of northern areas. Table 4 summarizes published studies in Italian populations.

**Table 4:** Prevalence of hypovitaminosis D in healthy children and adolescents living in Italy

Study	Period of enrolment	Number	M (%)	Age (range or as specified)	City/Region (Latitude)	% overweight	% obese	% receiving vitamin D	% not Caucasian	25(OH)D assay	Severe deficiency, % [25(OH)D < 10 ng/ml]	Deficiency, % [25(OH)D < 20 ng/ml]	Insufficiency, % [25(OH)D: 20–29.9 ng/ml]	Hypovitaminosis D, % [25(OH)D < 30 ng/ml]	Factors for associated with serum 25(OH)D levels
Lippi G et al. [69]	June 2004–June 2007	192	a	1 week-17.9 years	Verona (45°N)	a	a	a	a	CL	6.2 (< 11 ng/ml)	a	a	a	a
Marrone G et al. [70]	July 2009–June 2010	93	41.9	2–220 months	Udine (46°N)	a	11.8	33.3	14.0	CL	6.4 (< 5 ng/ml)	54.8	a	a	Ethnicity, BMI, seasonality
Pacifico L et al. [71]	Nov 2008-Mar 2009; Nov 2009-Mar 2010	452	46.7	b	Roma (41°N)	67.2 (overweight or obese)		a	0	CL	a	34.5 (< 17 ng/ml)	33.0 (17–27 ng/ml)	67.5 (< 27 ng/ml)	BMI
Lippi G et al. [72]	Oct 2008-Oct 2011	270	57.8	12.0–20.9 years	Parma (44°N)	a	a	a	a	CL	a	19.3	36.3	55.6	a
Mazzoleni S et al. [73]	Nov 2010-June 2012	58	69.0	1.1–15.3 years	Padua (45°N)	15.5	0	a	19.0	CL	a	50	27.6	77.6	a
Cadario F et al. [74]	June 2009-Sept 2009	62	a	1–3 days	Novara (45°N)	a	a	56.5 (mothers)	48.4	CL	46.3	75.6	a	a	Ethnicity, maternal supplementation
Vierucci F et al. [50]	Oct 2010-Sept 2012	652	49.7	2.0–21.0 years	Pisa (43°N)	18.9	23.0	0	5.7	RIA	9.5	45.9	33.9	79.5	Age, seasonality, ethnicity, BMI, sun exposure, sunscreen use
Bellone S et al. [75]	Jan 2010-Oct 2012	557	51.5	11.2 ± 0.1 years^c^	Novara (45°N)	79.7 (overweight or obese)		a	0	CL	a	Normal weight 31.8	Normal weight 39.9	Normal weight 71.7	BMI, waist and hip circumference
												Obese 44.4	Obese 36.7	Obese 81.1	
Franchi B et al. [76]	Jan 2010-Dec 2012	1374	53.9	0–16 years	Verona (45°N)	a	a	a	16.4	CL	a	Caucasian 44.2	Caucasian 30.6	Caucasian 74.8	Age, seasonality, ethnicity, gestational age, birth weight, BMI
														Not Caucasian 75.9–89.7	
												Not Caucasian 44.8–69.2			
													Not Caucasian 16.0–31.1		
Ciresi A et al. [77]	Jan 2011- Dec 2012	80^d^	72.5	4.3–16.0 years	Sicily (37°N)	a	a	a	a	CL	a	40.0	35.0	75.0	Seasonality
Stagi S et al. [78]	Sept 2010-Dec 2013	679	48.0	2.1–17.9 years	Florence (44°N)	24.7	4.3	0	0	CL	20.5	58.7	30.0	88.7	Age, seasonality, BMI, sun exposure, sunscreen use
Vierucci F et al. [79]	Oct 2010-Sept 2012	427	50.0	10.0–21.0 years	Pisa (43°N)	21.3	22.7	0	4.0	RIA	8.9	49.9	32.3	82.8	Age, seasonality, BMI, ethnicity, sun exposure, sunscreen use
Banzato C et al. [80]	Oct 2009-May 2010	32	65.6	7–16 years	Verona (45°N)	100.0		0	0	CL	31.3	84.4	a	a	a
Cadario F et al. [81]	Apr 2012-Mar 2013	533	a	1–3 days	Novara (45°N)	a	a	58.2 (mothers)	35.8	TM	33.3	85.4	12.5	97.9	Season of birth, ethnicity, maternal supplementation
Colao A et al. [82]	Oct 2012-Oct 2013	373	59.0	11–20 years	Campania (40°N)	19.8	26.8	0	a	CL	a	1.6	79.4	19.0	BMI, smoking, exercise performance
Prodam F et al. [83]	July 2009-Dec 2013	575	50.2	6–18 years	Novara (45°N)	28.2	71.8	0	0	CL	a	46.1	37.6	83.7	Age, BMI, waist circumference, seasonality, UVR, sun exposure
Di Nisio A et al. [84]	Oct 2013	108	100	11–14 years	Salerno (40°N)	45.4 (overweight or obese)		0	a	CL	a	a	a	83.3	BMI

*CL* chemiluminescence, *RIA* radioimmunoassay, *TM* tandem mass, *BMI* body mass index

^a^data not reported

^b^I 25(OH)D_3_ tertile: 11.5 ± 4.2 years; II 25(OH)D_3_ tertile 11.2 ± 4.3 years; III 25(OH)D_3_ tertile 11.0 ± 4.0 years (median ± interquartile range)

^c^mean ± standard error of mean

^d^children affected by growth hormone deficiency

## Sources of vitamin D and dietary reference values

Most of the vitamin D we synthesize (90%) starts with skin exposure to ultraviolet B radiation from the sun. Cutaneous vitamin D_3_ production is influenced by several factors, such as skin pigmentation, latitude, altitude, seasonality, daily timing of sun exposure, atmospheric pollution, percentage of skin area exposed, type of clothing, and sunscreen use. Children require less sunlight exposure than adults to produce sufficient quantities of vitamin D, both because of their higher body surface-area-to-volume ratio and of their increased capacity to produce vitamin D [85]. In Italy children are unable to synthesize vitamin D in the skin during late fall, winter months and early spring, even if sufficiently exposed to sunlight [50]. Thus, during this period an adequate vitamin D status can be maintained only from endogenous stores accumulated during previous summer or by exogenous supplementation.

Breast milk represents the best food to satisfy children’s nutritional needs, although it contains insufficient amount of vitamin D (< 50 IU/l) [86]. Vitamin D intake of formula fed infants varies according to the vitamin content (about 400 IU/l) and daily formula intakes. Considering water requirements, formula fed infants may receive 400 IU/day of vitamin D, which is adequate for the first year of life [10], only when they come to weigh 5 to 6 Kg. However, children are weaned by the time they reach this weight and this further reduces their daily milk consumption [1].

Most foods contain little amounts of vitamin D, with the exception of some fatty fish (Table 1), rarely eaten by children [9, 87]. Thus, dietary sources of vitamin D should not be considered significant for humans except some populations living at higher latitudes where fish, fish oil and fish eggs are frequently consumed [88]. In Italy, few commercial milks or yoghurts are supplemented with vitamin D and/or calcium. Nonetheless, despite the presence of this vitamin in fortified milk, amounts can be insufficient in respect of recommended intakes and needs. These foods, therefore, fail to represent an optimal solution for the prevention of vitamin-D deficiency among children and adolescents [50].

In the last years various Organizations and Societies [10, 11, 16–18, 25, 26, 89] revised dietary reference values of vitamin D in infants, children, and adolescents, as reported in Table 5. In 2016 the United Kingdom Scientific Advisory Committee on Nutrition (SACN) reviewed the evidence on vitamin D and health and recommended that serum 25(OH)D levels of all children and adolescents in the United Kingdom should not fall below 10 ng/ml (a so-called “population protective level”) at any time of the year to protect musculoskeletal health. Assuming minimal sunshine exposure, SACN recommended a safe intake of 340–400 IU/day of vitamin D for infants under 1 year of life, a safe intake of 400 IU/day for age 1 up to 4 years, and a reference nutrient intake of 400 IU/day for the United Kingdom population aged 4 years and above [25]. In 2016 also the European Food Safety Authority (EFSA) revised dietary reference values for vitamin D. Considering a serum 25(OH)D levels of 20 ng/ml as a suitable target value, under conditions of minimal cutaneous vitamin D synthesis EFSA recommended an adequate intake of 400 IU/day for infants aged 7–11 months and of 600 IU/day for children aged 1–17 years [26]. While there is sufficient global agreement in considering 400 IU/day as dietary reference value for vitamin D during the first year of life, recommended reference values for children and adolescents (1–18 years) slightly differ between Organizations and Societies, reflecting different approaches and methods applied to calculate them. However, various dietary reference values for vitamin D are useful to guide local strategies for vitamin D supplementation, but are not directly comparable. Particularly, the Endocrine Society guidelines focused on patients at risk for vitamin D deficiency, recommending a vitamin D daily requirement of 400–1000 IU in the first years of life and of 600–1000 IU from 1 up to 18 years [11].

**Table 5:** Dietary reference values of vitamin D in infants, children, and adolescents as proposed by various Organizations and Societies

Organization/Society	Year	Country/Countries	Dietary reference value for vitamin D	0–12 months, IU/day	1–18 years, IU/day
European Food Safety Authority [26]	2016	Europe	AI	400 (7–11 months)	600 (1–17 years)
Scientific Advisory Committee on Nutrition [25]	2016	United Kingdom	Safe Intake (< 4 years)	340–400	400
			RNI (4–18 years)		
Nordic Nutrition Recommendations [16]	2012	Denmark, Finland, Iceland, Norway, Sweden, Faroe Islands, Greenland, Åland Islands	RI	400	400
German Nutrition Society [17]	2012	Germany, Austria, Switzerland	AI	400 (infants)	800^a^ (children)
Health Council of the Netherlands [18]	2012	The Netherlands	AI	400	400
Italian Society of Nutrition [89]	2012	Italy	AI (< 12 months)	400 (6–12 months)	600
			PRI (1–18 years)		
Institute of Medicine [10]	2011	North America, Canada	AI (< 12 months)	400	600
			RDA (1–18 years)		
The Endocrine Society [11]	2011	Worldwide	Daily requirement^b^	400–1000	600–1000

*AI* Adequate Intake, the average observed daily level of intake by a population group of apparently healthy people that is assumed to be adequate

*Safe Intake*, a level or range of intakes considered to pose no risk of deficiency and below a level where there is a risk of undesirable effects

*RNI* Reference Nutrient Intake, the amount of a nutrient that is likely to meet the needs of 97.5% of the population

*RI* Recommended Intake, the amount of a nutrient that meets the known requirement and maintains good nutritional status among practically all (97–98%) healthy individuals in a particular life stage or gender group

*PRI* Population Reference Intake, the level of nutrient intake sufficient to satisfy the needs of almost all (97.5%) healthy subjects in one specific population group

*RDA* Recommended Dietary Allowance, the estimated intake capable of satisfying the needs of 97.5% of the population

^a^Adequate intake with missing endogenous synthesis of vitamin D

^b^Recommended requirements for subjects at risk of vitamin D deficiency

In accordance with the European Society for Paediatric Gastroenterology Hepatology and Nutrition [ESPGHAN] [19] and the European Academy of Pediatrics [30], we endorsed Tolerable Upper Intake Levels of vitamin D proposed by EFSA in 2012 (1000 IU/day for infants: 2000 IU/day for children ages 1 to 10 years; 4000 IU/day for children and adolescents ages 11 to 17 years) [90].

## Vitamin D supplementation

### 0–12 months

Vitamin D supplementation in the first year of life is essential to ensure an adequate vitamin D status and to prevent nutritional rickets. Indeed, newborns and infants are poorly exposed to sunlight, as the Section on Dermatology of the American Academy of Pediatrics (AAP) recommended that infants younger than 6 months of age should be kept out of direct sunlight and covered with appropriate protective clothing and hats [91]. As previously discussed, breast milk [86] and formula milk [1] contain insufficient amount of vitamin D to prevent its deficiency. Moreover, newborns from deficient mothers are at increased risk for vitamin D deficiency as cord blood and neonatal 25(OH)D levels highly correlate with maternal vitamin D status during gestation [92, 93]. The importance of vitamin D supplementation during the first year of life has been confirmed by the finding that children not receiving supplementation have reduced serum 25(OH)D levels, particularly if exclusively breastfed and during winter season [94–96].

Some studies evaluated the effect of daily vitamin D supplementation at variable dosages (ranging from 200 to 1600 IU/day) on vitamin D status in children during the first year of life [52, 62, 97–107]. The administration of 400 IU/day starting from birth was effective in maintaining serum 25(OH)D levels ≥30 ng/ml [52, 62, 97–99, 103–107]. On the contrary, vitamin D supplementation at higher dosages seemed to be associated with increased risk of hypervitaminosis D with hypercalciuria and hypercalcemia [62]. Daily vitamin D_2_ or D_3_ administration was equally effective in increasing serum 25(OH)D levels [108]. Only few studies evaluated the efficacy and safety of intermittent vitamin D supplementation during the first year of life, particularly in cases with low compliance [109–111], thus at present daily supplementation remains preferred.

Various international Scientific Societies agree to recommend vitamin D supplementation during the first year of life [9, 12–15, 19, 20, 22, 23, 27, 28, 30, 112–116]. Particularly, the Global Consensus on prevention of nutritional rickets recommended the administration of 400 IU/day of vitamin D for all infants from birth to 12 months of age, independently of their mode of feeding [28]. The same recommendation has been worldwide proposed also by an Expert Position Statement on vitamin D in childhood [1], the Department of Nutrition for Health and Development of the World Health Organization [116], the ESPGHAN [19], the European Academy of Pediatrics [30], and the vitamin D guidelines for United Arab Emirates [27] and Central Europe [20]. These Societies also agree that the administration of 400 IU/day is safe and effective to prevent rickets and ensure an adequate vitamin D status. The Endocrine Society recommended a daily intake of 400–1000 IU of vitamin D in children under 1 year of age at risk for vitamin D deficiency [11]. Regarding the preferred form of vitamin D for supplementation, the National Institute for Health and Care Excellence guideline recommended vitamin D drops in infants and young children [114], while other international Scientific Societies did not make any specific recommendation.

Despite this global agreement on vitamin D supplementation in the first year of life, several barriers to adherence still exist, such as reluctance of mothers to give their children daily supplementation, lack of knowledge about vitamin D actions and the risk of nutritional rickets, lack of awareness by health care professionals, assumption that both breast milk and formula milk provide sufficient vitamin D intake [116, 117].

In the present Consensus we recommend vitamin D supplementation in all newborns independently of the type of feeding, starting from birth and continuing throughout the first year of life. Infants born at term without risk factors for vitamin D deficiency should receive 400 IU/day of vitamin D, while in the presence of risk factors for vitamin D deficiency (Table 6) up to 1000 IU/day of vitamin D can be administered. We recommend against using vitamin D metabolites and their analogs (calcifediol, alfacalcidol, calcitriol, and dihydrotachysterol) for the routine vitamin D supplementation, as the administration of these compounds increases the risk of hypercalcemia and is not able to maintain and/or restore vitamin D stores [118, 119].

**Table 6:** Risk factors for vitamin D deficiency in the first year of life

• Non-Caucasian ethnicity with dark skin pigmentation	
• Inadequate diets (i.e. vegan diet)	
• Chronic kidney disease	
• Hepatic failure and/or cholestasis	
• Malabsorption syndromes (i.e. cystic fibrosis, inflammatory bowel diseases, celiac disease at diagnosis, etc.)	
• Chronic therapies: anticonvulsants, systemic glucocorticoids, antiretroviral therapy, systemic antifungals (i.e. ketoconazole)	
• Infants born from mothers with multiple risk factors for vitamin D deficiency, particularly in absence of vitamin D supplementation during pregnancy	

### Preterm

Preterm infants are at risk for calcium-phosphorus metabolism alterations, with possible development of osteopenia of prematurity [120]. As the majority of calcium and phosphate accretion and fetal bone mineralization occur during the third trimester of pregnancy, preterm infants are deprived of the physiological mineral intrauterine supply, with consequent impaired bone mineralization and increased fracture risk. Very low birth weight (VLBW) infants (birth weight < 1500 g) are at significant risk of osteopenia due to the frequent administration of drugs that adversely affect bone mineralization (steroids, methylxanthines and diuretics) and prolonged period of immobilization and total parenteral nutrition [121]. At present, the exact timing and proportion of vitamin D-dependent absorption of calcium and phosphorus in preterm infants is unknown [122]. However, after 24 weeks gestation maternal 25(OH)D crosses the placenta and is metabolized to 1,25(OH)_2_D for endocrine and paracrine actions [123]. In addition to optimize bone mineralization during fetal life, vitamin D status has been associated with acute respiratory morbidity in preterm infants born < 32 weeks gestation [124].

Relatively few international Scientific Societies gave recommendations on vitamin D supplementation in preterm infants. In 2013 the AAP recommended a daily vitamin D intake of 200–400 IU for VLBW infants, as their smaller size may lead to a lower need for vitamin D to ensure adequate serum 25(OH)D levels. Vitamin D intake should be increased to 400 IU/day (up to a maximum of 1000 IU/day) when weight exceeds 1500 g and the infant tolerates full enteral nutrition [122]. Differently, considering the high prevalence of vitamin D deficiency during pregnancy, the ESPGHAN recommended for preterm infants a vitamin D intake of 800–1000 IU/day during the first months of life to rapidly correct fetal low serum 25(OH)D levels [125]. Other authors and guidelines for Central Europe recommended vitamin D supplementation in preterm infants at higher dosages (400–1000 IU/day) than those suggested for healthy term newborns [1, 9, 20, 126], but such an intake should not be prolonged over the theoretical term (40 weeks of post-conceptional age) due to the potential risk of vitamin D intoxication [126].

Preterm infants may receive vitamin D from various sources, such as parenteral nutrition, fortified human milk or preterm infant formula, but it has been calculated that without supplementation they do not receive 400 IU/day of vitamin D until reaching a weight of 2–2.5 Kg [123] or only 4 weeks after birth [127]. However, total vitamin D intake from supplementation and feeding should be assessed to avoid excess, particularly in VLBW infants.

Some studies evaluated the effect of vitamin D supplementation at different doses in preterm infants of various gestational age and birth weight [124, 128–146]. Vitamin D supplementation at 400 IU/day has been generally retained safe and effective in maintaining adequate serum 25(OH)D levels in preterm infants [131, 132, 135, 139–141]. Studies that evaluated vitamin D supplementation at 200 IU/day gave conflicting results [128, 129, 134]. Finally, some studies evaluated the effect of supplementation at higher doses (800–1000 IU/day) [133, 135–137, 140–146]. Cho et al. recently recommended vitamin D supplementation at 800 IU/day to enhance vitamin D status during early hospitalization in VLBW infants with serum 25(OH)D levels < 10 ng/ml at birth [142]. Mathur et al. found that supplementation at 1000 IU/day for 6 weeks was more effective than 400 IU/day in maintaining serum 25(OH)D levels with a lower incidence of skeletal hypomineralization and better growth [144]. However, other authors did not find any difference in clinical outcome or in bone accrual in preterm infants receiving supplementation at higher doses [135–137, 143] and advised to avoid prolonged supplementation at 1000 IU/day for the risk of hypervitaminosis D [143, 145, 146]. Particularly, Fort et al. suggested for extremely low gestational age newborns an initial vitamin D supplementation at 800 IU/day for 1–2 weeks to restore serum 25(OH)D levels followed by a lower dosage (200 IU/day) [143].

In the present Consensus we recommend a total daily vitamin D intake of 200–400 IU (including the amount administered through parenteral nutrition, fortified breast milk, and preterm formula) for preterm infants with a birth weight < 1500 g. Vitamin D supplementation at 400–800 IU/day is recommended for VLBW infants when they reach a weight ≥ 1500 g and full enteral nutrition, and for preterm infants with a birth weight ≥ 1500 g. After a post-conceptional age of 40 weeks, recommendations for vitamin D supplementation are equal to those for healthy term infants.

### 1–18 years

The promotion of an adequate vitamin D status is important for older children and adolescents, as nutritional rickets may develop during the entire pediatric age [28] and vitamin D deficiency may negatively affect bone health [25, 26]. Various studies evaluated different regimens of vitamin D supplementation, but comparison of results is complex due to heterogeneity in vitamin D administration (dose, interval, and length of supplementation) and population enrolled (age, gender, ethnicity, body mass index, latitude of the country of residence, season of enrolment, and basal vitamin D status).

Most of the studies evaluated daily vitamin D supplementation at doses ranging from 200 to 1000 IU/day [78, 147–164]. Supplementation at 400 IU/day for variable length (up to 12 months) was usually insufficient in raising serum 25(OH)D levels > 30 ng/ml [78, 147–149, 151–153, 160–162], particularly in subjects with vitamin D deficiency. A recent RCT performed during winter showed that a vitamin D intake up to 800 IU/day was required by white Danish children (4–8 years) to maintain serum 25(OH)D > 20 ng/ml. Particularly, subjects receiving 800 IU/day for 20 weeks increased their 25(OH)D levels from 23.2 ng/ml to 30.3 ng/ml [161]. Another RCT demonstrated that white UK adolescents (14–18 years) required higher intake of vitamin D (up to 1200 IU/day) during winter to achieve 25(OH)D concentration > 20 ng/ml in 97.5% of cases. Indeed, children and adolescents supplemented with 800 or 1000 IU/day for 20 weeks increased their 25(OH)D levels but frequently remained insufficient [156–158, 162–164], while supplementation at 300 IU/day for 7 weeks did not result efficacious in younger children [163]. A few studies evaluated intermittent regimens of supplementation (weekly, monthly, every 2–6 months) in older children and adolescents, with conflicting results [165–175]. Intermittent vitamin D administration may be considered in case of reduced compliance with daily supplementation, but actual evidence is insufficient to recommend a preferred doses and interval.

Several international Societies recommended vitamin D supplementation in children older than 1 year and adolescents with risk factors for vitamin D, such as reduced sun exposure or dark skin pigmentation [8, 9, 11, 18, 19, 23, 28, 30, 114]. Particularly, the ESPGHAN reinforced that first of all a healthy lifestyle associated with a normal body mass index and including a healthy diet with vitamin D-containing foods and adequate outdoor activities should be promoted in healthy children and adolescents [19]. Moreover, pediatricians should periodically evaluate vitamin D intake from diet and supplements [23, 28]. Considering the Italian Child Health Care System organization, family pediatricians may anamnestically evaluate vitamin D intake of children and possible risk factors for deficiency during periodic health check-ups [176].

On the contrary, other Societies systematically recommended vitamin D supplementation in children and adolescents during winter months [13, 20, 115] or throughout the whole year if reduced sun exposure during summer [20, 115]. Following the publication of SACN review of the evidence on vitamin D and health [25], Public Health England advised that UK children aged 1 to 4 years should receive vitamin D supplementation at 400 IU/day, and older children and adolescents should take a daily supplement containing 400 IU of vitamin D in autumn and winter to protect bone and muscle health because it is difficult to meet this intake from dietary sources. Public Health England also recommended that individuals with darker skin and people with reduced sun exposure should receive vitamin D supplementation throughout the year [115].

Adolescents are at increased risk for vitamin D deficiency [79], thus the Society for Adolescent Health and Medicine recommended continuous vitamin D supplementation (600 IU daily for healthy adolescents, and at least 1000 IU daily for adolescents at risk for vitamin D deficiency or insufficiency) in addition to vitamin D received through the diet or via sun exposure [21].

Variation of sunlight efficacy in promoting skin vitamin D synthesis (depending on season and latitude) and local factors related to sunlight exposure (i.e. cultural habits) should be taken into account when considering supplementation [30, 177, 178]. For example, Arab Emirates guidelines recommended vitamin D supplementation between May and October because Arabian people avoid sun exposure during summer due to excessive heat [27]. Regarding Italy (latitude 35°29′24″-47°5′31″), an in vitro study showed that no vitamin D is produced as a result of sun exposure at the latitude of Pisa (43°43’N) from November to February [179], confirming that sunlight-derived vitamin D production is ineffective for at least 1 month during the year in countries placed between 23.5° and 66.5° of latitude [180]. Subsequent Italian cross sectional studies enrolling children and adolescents living in the north-western area of Tuscany, Central Italy (latitude between 43°N and 44°N) not receiving vitamin D supplementation confirmed seasonal variability in serum 25(OH)D levels, with lower concentrations during late winter-early spring months (February–April), with negligible amount of vitamin D obtained from diet [50, 79], according to other Italian pediatric studies [70, 76–78, 83]. These data suggest that wintry vitamin D status depends on the amount of vitamin D produced and stored during the previous summer [181]. Finally, a recent cross sectional study showed high prevalence of vitamin D deficiency and insufficiency (40.3% and 33.5%, respectively) among internationally adopted children at their first clinical evaluation in Italy [182].

In the present Consensus we recommend vitamin D supplementation in children and adolescents with risk factors for vitamin D deficiency (Table 7), at doses ranging from 600 IU/day (i.e. in presence of reduced sun exposure) up to 1000 IU/day (i.e. in presence of multiple risk factors for vitamin D deficiency). In cases of poor compliance, supplementation with intermittent dosing (weekly or monthly doses for a cumulative monthly dose of 18000–30000 IU of vitamin D) can be considered, starting from children aged 5–6 years and particularly during adolescence. Considering the results of Italian studies, we suggest vitamin D supplementation from the end of fall to the beginning of spring (November–April) in children and adolescents with reduced sun exposure during summer. Continuous supplementation should be reserved to children with permanent risk factors for vitamin D deficiency. Individuals on anticonvulsants, oral corticosteroids, antimicotics and antiretroviral drugs should receive at least 2–3 times more vitamin D than the daily requirement recommended for age, in agreement with the Endocrine Society [11] and the AAP [23]. As reported for infants in the first years of life, we recommend against using vitamin D metabolites and their analogs (calcifediol, alfacalcidol, calcitriol, and dihydrotachysterol) for the routine vitamin D supplementation.

**Table 7:** Risk factors for vitamin D deficiency between 1 and 18 years of age

• Non-Caucasian ethnicity with dark skin pigmentation	
• Reduced sunlight exposure (due to lifestyle factors, chronic illness or hospitalization, complex disability, institutionalization, covering clothing for religious or cultural reasons) and/or constant use of sunscreens	
• International adoption	
• Obesity	
• Inadequate diets (i.e. vegan diet)	
• Chronic kidney disease	
• Hepatic failure and/or cholestasis	
• Malabsorption syndromes (i.e. cystic fibrosis, inflammatory bowel diseases, celiac disease at diagnosis, etc.)	
• Chronic therapies: anticonvulsants, systemic glucocorticoids, antiretroviral therapy, systemic antifungals (i.e. ketoconazole)	

At present, population screening for vitamin D deficiency in healthy individuals is not recommended. Indeed, serum 25(OH)D evaluation should be reserved to subjects at risk for vitamin D deficiency, but indications for 25(OH)D measurement significantly vary among different societies (Table 8) [9, 11, 13, 15, 20–23, 27, 30]. We recommend against routine 25(OH)D testing in children and adolescents, suggesting to limit serum 25(OH)D levels evaluation in children and adolescents with multiple risk factors for vitamin D deficiency. Particularly, vitamin D status should be monitored at least yearly in subjects that require supplementation during the whole year because affected from pathological conditions or receiving drugs affecting vitamin D metabolism (Table 7) (see “Extraskeletal actions of vitamin D” for specific recommendations).

**Table 8:** Indications for 25(OH)D evaluation (besides rickets) in children and adolescents as proposed by various Organizations and Societies

Organization/Society, year of publication	PES, 2008 [9]	ES, 2011 [11]	FCN, 2012 [15]	French Soc. of Pediatrics, 2012^a^ [13]	Australia - New Zealand, 2013 [22]	Central Europe, 2013 [20]	SAHM, 2013 [21]	AAP, 2014 [23]	Arab Emirates, 2016 [27]	EAP, 2017 [30]
Frequent/low trauma fractures and/or low BMD	X	X	X			X	X	X^b^	X	
Calcium/phosphate metabolism abnormalities						X			X	
Immobilization/disabilities					X		X	X	X	
Dark skin pigmentation	X^c^	X^d^	X	X	X		X			X
Reduced sun exposure	X			X	X		X			X
Athletes (indoor sports)			X							
Children institutionalized									X	X
Constant use of sunscreens							X			
Obesity		X	X^e^	X	X		X		X	
Inadequate diets (e.g. vegan)				X			X			X
Elimination diets (e.g. cow/s milk allergy), eating disorders						X		X	X	
On anticonvulsants	X	X	X	X	X	X	X	X	X	X
On chronic glucocorticoids	X	X	X			X	X	X	X	
On HIV medications		X	X			X	X		X	
On antifungals (e.g. ketoconazole)		X	X			X			X	
On rifampicin				X	X					
Malabsorption syndromes	X	X	X	X	X	X	X	X	X	X
Chronic kidney disease		X	X	X	X	X	X	X	X	X
Nephrotic syndrome				X						
Hepatic failure and/or cholestasis		X	X	X	X	X	X		X	X
Granulomatous disorders (e.g. tuberculosis)		X	X			X			X	
Amenorrhea							X			
Cancer (different types)		X^f^				X		X	X	
Hepatitis C^g^						X			X	
Recurrent acute lower respiratory tract infections^g^						X			X	
Atopic dermatitis^g^						X			X	
Atopic Asthma^g^						X			X	
Autoimmune diseases^h^						X		X^i^	X	
Cardiovascular diseases (especially hypertension)						X			X	
Metabolic syndrome, type 2 diabetes									X	

*PES* Pediatric Endocrine Society, *ES* Endocrine Society, *FCN* Federal Commission for Nutrition (Switzerland), *SAHM* Society for adolescent health and medicine, *AAP* American Academy of Pediatrics, *EAP* European Academy of Pediatrics

^a^suggested to guide vitamin D prescription in individuals with underlying risks requiring continuing supplementation all year long, if necessary

^b^genetic conditions (osteogenesis imperfecta, idiopathic juvenile osteoporosis, Turner syndrome), endocrine conditions (Cushing syndrome, hypogonadism, hyperthyroidism, hyperparathyroidism, growth hormone deficiency, diabetes mellitus) associated with reduced bone mass

^c^living at higher latitudes in the winter and spring months

^d^African-American and Hispanic children

^e^obese children with additional risk factors/symptoms for vitamin D deficiency

^f^ some lymphomas

^g^patients admitted to hospital

^h^multiple sclerosis, psoriasis, rheumatoid arthritis, dermatomyositis, systemic lupus erythematosus

^i^ systemic lupus erythematosus, juvenile idiopathic arthritis, juvenile dermatomyositis as associated with reduced bone mass

## Skeletal actions of vitamin D

### Nutritional rickets

Rickets is characterized by defective mineralization of developing bone tissue and reduced or absent endochondral ossification of the growth plate, with subsequent deformation [183, 184]. Nutritional rickets is caused by vitamin D deficiency and/or low calcium intake in children. Despite a significant decrease in the incidence and prevalence of nutritional rickets during the twentieth century, new cases are still reported worldwide, both in developing and industrialized countries [185, 186].

Children of immigrants living in industrialized countries are at increased risk of rickets because they usually present several risk factors for vitamin D deficiency such as prolonged breastfeeding without vitamin D supplementation, increased skin pigmentation, reduced sun exposure due to cultural habits (i.e. veiling), and reduced intestinal calcium uptake due to an excessive intake of high-phytates foods [187]. At present, no exact serum 25(OH)D threshold has been defined below which nutritional rickets may develops [33]. A recent global consensus recommendations on prevention and management of nutritional rickets defined vitamin D deficiency as serum 25(OH)D <  12 ng/ml, considering the increased incidence of rickets with serum 25(OH)D levels under this threshold [28]. Similarly, in 2016 the SACN reported that the risk of nutritional rickets increased for serum 25(OH)D levels < 10 ng/ml [25].

Nutritional rickets diagnosis is based on the evaluation of clinical, radiological and biochemical findings, as reviewed elsewhere [9, 183, 188]. Rickets typically develops towards the end of the first year of life and in the course of the second year of life. Subsequently, the clinical signs of vitamin D deficiency (i.e. rickety wrists and ankles, rachitic rosary, Harrison’s sulci, lower limb deformities) become more subtle. Particularly, adolescents may develop non-specific symptoms, such as lower limb pain or difficulties in climbing stairs, because of proximal myopathy secondary to vitamin D deficiency [189]. Nutritional rickets may be associated also with extraskeletal manifestations, such as muscular hypotonia, delayed motor development, and increased risk of respiratory infections. Moreover, vitamin D deficiency may determine hypocalcaemia that may be asymptomatic, latent or symptomatic possibly with acute onset (seizures, syncope, laryngospasm, bronchospasm, tetanus, paresthesia, tremors, muscular cramps, dilated cardiomyopathy) [188]. At X-rays osteopenia of the long bones may be the earliest radiological sign of rickets. Subsequently, a fraying and cupping deformity of metaphyses following the proliferation of uncalcified cartilage and osteoid tissue is usually observed [190]. If nutritional rickets is suspected, biochemical investigations [serum 25(OH)D, PTH, alkaline phosphatase, calcium, and phosphorus levels] and X-rays evaluation of metaphyseal sites (wrists and ankles) are recommended [188]. On the contrary, the evaluation of serum 1,25(OH)_2_D and bone turnover markers is not useful to pose diagnosis.

Vitamin D administration represents the main treatment of nutritional rickets [183, 188], and some age-dependent regimens have been proposed [9, 28, 183]. Oral treatment is preferred as it more rapidly restores serum 25(OH)D levels than intramuscular treatment [28]. The global consensus on nutritional rickets recommended the administration of 2000 IU/day of vitamin D in patients aged less than 1 year, 3000–6000 IU/day in patients aged 1 to 12 years and 6000 IU/day in patients older than 12 years for a minimum of 3 months, even if some children may require a longer treatment duration [28]. Intermittent administration of vitamin D may be a reliable alternative to daily administration, particularly in cases with low compliance. Indeed, Munns et al. recommended a single dose of 50000 IU in children age 3 to 12 months, 150000 IU in children aged 1 to 12 years, and 300000 IU in adolescents > 12 years of age. When single large doses are used, vitamin D_3_ is preferable compared to vitamin D_2_ because the former has a longer half-life [28]. Monthly oral administration of 100000 IU for three consecutive months was demonstrated to be safe and effective to treat nutritional rickets in immigrant children and adolescents living in Italy [187]. The administration of a single large dose of vitamin D > 300000 IU is not recommended, because may cause high risk of vitamin D intoxication with hypercalciuria and hypercalcemia, as confirmed by recent studies [191–195]. After rickets healing, vitamin D supplementation should continue according to age (at least 400 IU/day in the first year of life and 600 IU/day from 1 to 18 years) [11, 23, 28, 183, 188]. Besides vitamin D, calcium is also important to treat nutritional rickets, even in absence of hypocalcemia. Munns et al. recommended oral calcium administration of 500 mg/day in conjunction with vitamin D regardless of age or weight [28], while Misra et al. recommended 30–75 mg/kg/day of elemental calcium in 3 divided doses, starting at a higher dose and weaning down to the lower end of the range over 2–4 weeks [9]. Calcium should be administered intravenously in presence of acute, symptomatic hypocalcaemia. Calcium supplementation is important to prevent “hungry-bone” syndrome (hypocalcemia secondary to an increase in bone mineralization as PTH levels normalize during vitamin D treatment) [9]. Vitamin D metabolites and their analogs (calcifediol, alfacalcidol, calcitriol, and dihydrotachysterol) are not recommended for treatment of nutritional rickets. Particularly, the use of 1α-hydroxylated metabolites of vitamin D does not restore vitamin D levels, and may determine supraphysiological levels of 1,25(OH)_2_D with increased risk of hypercalcemia [183, 188]. The administration of 1α-hydroxylated metabolites of vitamin D may be considered in nutritional rickets associated with acute hypocalcemia or hypocalcemic cardiomyopathy [9, 183].

### Bone health

Bone mass acquisition is influenced by both genetics and lifestyle-related factors, such as vitamin D status, physical activity and calcium intake [23, 53]. Vitamin D contributes significantly to bone mineralization by promoting intestinal calcium and phosphorus reabsorption. Moreover, vitamin D stimulates skeletal calcium and phosphorus and renal calcium reabsorption. Besides the direct regulation of calcium-phosphorus metabolism, vitamin D also indirectly promotes bone mass accrual stimulating the development of muscle tissue [196–198]. Bone mass acquisition starts during fetal life and continues throughout the entire pediatric age until young adulthood with the achievement of peak bone mass (PBM), that is the total amount of bone mass acquired when accrual plateaus after completion of growth and development [199]. As bone mass tracks during childhood and adolescence, bone status during pediatric age is a strong predictor of bone status in young adulthood [200].

As discussed before, vitamin D supplementation during the first year of life is essential to prevent nutritional rickets occurrence. A few studies evaluated the relationship between vitamin D status and bone mass during this period of life [52, 62, 97, 104, 201–203]. Among these, three Italian studies assessed bone mineral status in infants using quantitative ultrasound [201–203], suggesting that vitamin D supplementation is important to provide an adequate bone development, particularly in exclusively breastfed infants. On the contrary, studies using dual-energy X-ray absorptiometry (DXA) or peripheral quantitative computed tomography (pQCT) failed to demonstrate an association between serum 25(OH)D levels and bone mass parameters in the first year of life [52, 104]. Moreover, studies comparing supplementation with placebo or different regimens of vitamin D supplementation (up to 1600 IU/day) did not find any difference in infant bone mass at 3 months [104], 6 months [97], 1 year [62] or 3 years of life [204]. Interestingly, higher vitamin D status from infancy through to 3 years of age was associated with leaner body composition [205]. Thus, vitamin D supplementation during infancy is important to optimize bone mass acquisition and body composition, but high doses are not recommended.

Several studies evaluated the association between vitamin D status and bone mass in children and adolescents, usually searching for a correlation between actual serum 25(OH)D levels and bone mass. Results of these studies are heterogeneous, because some demonstrated that vitamin D status was significantly related to bone mass [41, 42, 54, 206–208] while others did not find any association [44, 209–211]. Vitamin D status seems particularly important for bone health during adolescence. Indeed, duodenal expression of 25-hydroxyvitamin D_3_-1α-hydroxylase is higher in adolescents than in children and adults, representing a metabolic adaptation to promote dietary calcium absorption for the growing bone [212]. A recent study confirmed that serum 25(OH)D levels correlated with bone density and bone quality pQCT parameters in adolescents [213]. Moreover, vitamin D status was demonstrated to be a significant determinant of PBM in young adults [214, 215].

In 2010 a Cochrane meta-analysis of 6 randomized controlled trials (RCTs) [148, 153, 166, 216–218] evaluated the effect of vitamin D supplementation on BMD in healthy children and adolescents (age 8–17 years, 541 subjects receiving vitamin D and 343 receiving placebo) [219]. Overall, meta-analysis showed a non-significant effect of vitamin D supplementation on BMD at any site. However, the effect of supplementation became significant on total body BMC and lumbar spine BMD by dividing the sample into two groups, depending on vitamin D status [25(OH)D levels < 14 ng/ml vs. ≥ 14 ng/ml], suggesting that vitamin D supplementation may result in a clinically significant increase in bone mass in subjects with vitamin D deficiency. In 2016 the National Osteoporosis Foundation applied an evidence based grading system to describe the strength of available evidence on modifiable lifestyle factors that may influence the acquisition of PBM, reporting moderate evidence (grade B) for vitamin D [220]. This systematic review selected 1 prospective study [221], 3 cross-sectional studies [42, 222, 223], and 8 RCTs of vitamin D supplementation. Five RCTs were already evaluated by the Cochrane meta-analysis [148, 153, 166, 216, 217], while the remaining 3 studies were published in 2010 [147, 224, 225]. Particularly, 4 RCTs provide evidence for a beneficial effect of vitamin D supplementation on bone mineral accrual [148, 166, 216, 224], mainly in subjects with vitamin D deficiency. In 2016, SACN and EFSA revised dietary reference values for vitamin D reporting an increased risk of adverse musculoskeletal health outcomes at serum 25(OH)D levels in the range of deficiency, but with different thresholds (< 10 ng/ml and <  20 ng/ml, respectively) [25, 26]. Interestingly, a recent study showed that the beneficial effect of vitamin D on hip bone mass in Lebanese adolescent girls persisted 1 year after discontinuation of supplementation [226]. At present, some unanswered questions remain [critical times during which supplementation may be most effective, regimen and length of supplementation (continuous or intermittent), gender difference] [227], thus vitamin D supplementation to optimize bone mass acquisition should be reserved for children at risk for deficiency.

Maternal vitamin D status during pregnancy may significantly influence fetal and neonatal bone mass, as recently confirmed by the SACN [25]. Particularly, fetal bone growth was associated with maternal serum 25(OH)D at 26 weeks [228] and 34 weeks of gestation [229]. Newborns from mothers with serum 25(OH)D levels < 17 ng/ml (median value of the individual means for maternal blood samples collected during the first trimester and 2 days postpartum) had higher tibia BMC and larger cross-sectional area, as assessed by pQCT at 10 days postpartum. These results confirmed that maternal vitamin D status may affect bone mineral accrual during the intrauterine period and influence bone size [230]. Moreover, postnatal vitamin D supplementation (follow-up 14 months) may only partly eliminate the differences in bone variables induced by maternal vitamin D status during the fetal period [231]. Recently an English RCT (MAVIDOS) failed to demonstrate an effect of maternal vitamin D supplementation (1000 IU/day from 14 week of gestation until delivery) on offspring bone mass assessed within 2 weeks of birth by DXA. However, secondary analysis showed a benefit for neonatal whole-body BMC (increased by almost 10% vs. placebo) with supplementation for deliveries during winter [232]. Women who gave birth in winter had a mean 25(OH)D concentration of 12 ng/ml at 34 weeks of gestation, suggesting a threshold at which supplementation may significantly affect neonatal bone mass [233]. Another smaller RCT on 50 newborns failed to demonstrate an effect of vitamin D supplementation during pregnancy (2000 IU/day from 26 to 28 weeks until childbirth) on infant bone mass measured at 23 ± 10 days by DXA [234].

Uncertain evidence exists on the relationship between maternal gestational 25(OH)D levels and offspring bone mass later in life (from 12 months to 10 years) [235–239]. Several variables may justify this discrepancy, such as different enrolled populations, various regimens of vitamin D supplementation, and age at bone mass assessment. However, the most recent study suggested that vitamin D status during childhood might be more relevant for bone health that maternal 25(OH)D levels during fetal life [237].

## Extraskeletal actions of vitamin D

### Respiratory infections

Vitamin D has complex immunoregolatory properties, exerted by modulating both innate and adaptive immunity and regulating the inflammatory response. A relationship between vitamin D status and the incidence or the severity of respiratory infections in children has been found in many observational studies, most with a case-control or cross sectional design, both in developing and in westernized countries. Some systematic reviews have addressed this topic as well [240–242]. The link between severe vitamin D deficiency and susceptibility to respiratory infections is prototypically represented by the high respiratory morbidity in children with rickets [243, 244]. Children with rickets are more likely to develop pneumonia or poor outcomes after lower respiratory infections [244, 245]. Other than pneumonia, some data also indicate that vitamin D deficiency might be a risk factor also for undifferentiated viral infections [246], recurrent pharyngotonsillitis [247], otitis media [248], bronchiolitis [249] and viral wheezing [250]. Some authors also postulated that the lower vitamin D production in winter might contribute to the marked seasonality of epidemic influenza [251]. Obviously, the more robust evidences on the association between vitamin D deficiency and respiratory infections stem from prospective studies. Camargo et al., after adjusting for season of birth, found that newborns with vitamin D cord blood levels < 10 ng/ml had a two-fold (odds ratio: 2.16; 95% CI 1.35–3.46) increased risk of respiratory infections by 3 months of age as compared to newborns with 25(OH)D concentrations > 30 ng/ml [252]. Belderbos et al. also demonstrated that cord blood vitamin D levels < 20 ng/ml were associated with a sixfold (95% CI: 1.6–24.9) increased risk of respiratory syncytial virus lower respiratory tract infections in the first year of life as compared with concentrations ≥30 ng/ml [249]. Science et al. in a cohort of 743 Canadian children aged 3–15 years prospectively followed for 6 months found that 25(OH)D D levels < 20 ng/ml increased the risk of laboratory confirmed viral infections by 70% [246]. Other studies have found an inverse association between vitamin D concentrations in pregnancy and the risk of respiratory infections in newborns or children during the first 3 years of life [253, 254]. However, not all studies have confirmed these associations [255, 256]. For example, no relationship has been found in children and adults between baseline vitamin D concentrations and influenza virus infection [256].

Taken together, all these data indicate that vitamin D status might have some influence on conditioning the incidence and severity of some but not all type of respiratory infections and that more information are required before drawing any firm conclusion on this topic. A recent literature review supports a role for vitamin D deficiency only for tuberculosis, recurrent acute otitis media and severe bronchiolitis [242]. Furthermore, some role from VDR polymorphisms or other genetic factors might play a role in determining the influence of vitamin D status on respiratory morbidity [257, 258].

Several studies, mostly in adults, have also addressed the question whether vitamin D supplementation can prevent or reduce the severity of upper or lower respiratory infections. Systematic reviews with or without meta-analysis [241, 259–266] also focused on this topic. Of those, four included only studies in children [241, 263, 264, 266]. All but two [259, 261] of the aforementioned reviews concluded that published studies do not indicate a protective effect of vitamin D supplementation on the prevention of acute respiratory infections in healthy individuals. Some authors also indicated a possible publication bias [261, 266]. However, a well conducted study, not included in the above cited meta-analysis, on a selected population of otitis prone children, found that vitamin D administration (1000 IU/day) reduces the risk of uncomplicated acute otitis [267].

A recent meta-analysis of 25 RCTs (total 11,321 participants, aged 0–95 years) showed that vitamin D supplementation was safe and protected against acute respiratory tract infections overall (adjusted odds ratio 0.88). Subjects with severe deficiency [25(OH)D <  10 ng/ml] and those receiving daily or weekly doses rather than bolus dose had greater benefits [268], although the indication for this condition is still debated [269, 270]. Indeed, a more recent Canadian RCT showed that daily administration of 2000 IU of vitamin D did not reduce overall wintertime upper respiratory tract infections among healthy children aged 1–5 years compared with supplementation with 400 IU/day. Thus, the results of this study do not support the routine use of high-dose vitamin D supplementation in children for the prevention of viral upper respiratory tract infections [271]. Finally, limited data are available on vitamin D supplementation in pregnancy and the risk of respiratory infections in the offspring [272, 273].

### Other infections

An association between serum 25(OH)D levels and several types of pediatric infections has been reported. In a Turkish study including 82 children with urinary tract infection and 64 healthy controls lower serum 25(OH)D concentrations were evaluated, observing lower levels in infected children [274]. Moreover, VDR gene polymorphisms can be important for susceptibility to urinary tract infection and renal scar formation [275]. In an USA study a similar association was observed between skin or soft tissue infections by *Staphylococcus aureus* strains and vitamin D levels [25(OH)D < 30 ng/ml] in 202 children [276]. Other authors reported similar findings in children with acute diarrhea [277], otitis [277], rotavirus infection [278], malaria [279], leishmaniosis [280], hepatitis C [281], or sepsis [282]. However, it is unclear whether the observed vitamin D deficiency/insufficiency is to be considered a consequence of the infection itself or if it plays a role in determining susceptibility to infections or the severity of the disease. Interpretations of study results should also consider the different settings, the nutritional status of the enrolled children and the prevalence of co-infections.

Few reports exist regarding the benefit of vitamin D supplementation in children with specific infections. In a large randomized study in Afghanistan involving 3000 children with acute diarrhea vitamin D supplementation has been associated with improvement in clinical parameters [283]. The interpretation of results conducted in human immunodeficiency virus (HIV) infected patient is extremely complex. The vitamin D status can be influenced by the infection itself but also by antiretroviral therapy. Moodley et al. evaluated more than 900 infected children and observed that vitamin D-related host genetic variants may alter the availability and activity of vitamin D and are associated with risk of HIV disease progression in children [284]. In another study in Tanzania, serum 25(OH)D levels were evaluated in 884 pregnant women and, subsequently, in their infants. Low maternal 25(OH)D levels (< 32 ng/ml) were associated with a 46% higher risk of mother to child transmission of HIV. Moreover, children born from women with a low 25(OH)D level had a 61% higher risk of dying during follow-up [285]. In a large French study vitamin D deficiency was more frequent in 113 children with HIV than in 54 healthy controls [286]. Similar results were observed by Rustein et al. in USA [287]. An Italian RCT was performed to test whether vitamin D_3_ supplementation (oral 100000 IU every 3 months for 4 doses) could improve vitamin D status and affect the T-cell phenotype in HIV-infected patients aged 8 to 26 years with serum 25(OH) D < 30 ng/ml. Supplementation increased 25(OH)D and 1,25(OH)_2_D concentrations and decreased PTH levels but had no effect on CD4+ T-lymphocyte count. However, it was associated with changes in CD4+ T-lymphocyte phenotype [288]. In a pilot study in Botswana, vitamin D_3_ supplementation (4000–7000 IU/day for 12 weeks) was safe and improved vitamin D status, growth and HIV status [289]. Similarly, Dougherty et al. confirmed that a 7000 IU/day D_3_ supplementation was safe and effective in HIV infected children and young adults [290].

Tuberculosis is the infection probably more deeply studied at this regard [291–294]. Several studies investigated a possible relationship between vitamin D deficiency and tuberculosis infection in children [295], but literature results are discordant. Significant association between active tuberculosis and vitamin D deficiency [25(OH)D levels < 20 ng/ml] has been evidenced in an Australian study including 91 children with latent or active tuberculosis and 236 controls [296], similarly to one UK study [297]. A more recent large study on 996 children serum 25(OH)D levels < 20 ng/ml were more frequently observed in children with tuberculosis infection than in healthy controls [298]. Conversely, such association was not confirmed in a smaller Indian study [299]. Moreover, a large meta-analysis failed to demonstrate that vitamin D supplementation may be beneficial in children with tubercular infection [300]. Chiappini et al. did not find an association between tubercular infection and vitamin D status in a large population of internationally adopted children, although results demonstrated a high prevalence (about 75%) of hypovitaminosis D in this population [182]. In the same study parassitosis was not related to serum 25(OH)D levels. The same authors had previously demonstrated a relation between tubercular disease and vitamin D status [301]. However, it should be noticed that only two internationally adopted children had active tubercular disease, possibly explaining the discrepancy with respect to previous results.

These results are in line with those reported by Grobler et al. in a Cochrane review in 2016. According to this review, although blood levels of some vitamins may be low in patients starting treatment for active tuberculosis, there is currently no reliable evidence that routine supplementation at or above recommended daily amounts has clinical benefits [302].

### Asthma

Vitamin D status has also been linked with asthma development and control [303]. Recently, Litonjua showed that vitamin D has both in utero and post-natal effects on lung development and immune system development and function [304]. Adverse exposures in this critical period, such as low levels of serum 25(OH)D, might lead to developmental changes including reduced lung and airway growth and could therefore be a major importance for the development of asthma. Vitamin D appears to affect innate and adaptive immune system development through lymphocyte activation and proliferation, and T-helper cell differentiation [305]. Several studies showed that vitamin D deficiency in utero and in early life was associated with an increase in Th2 lymphocyte cells and a reduction in T regulatory cells and production of interleukin (IL)-10, which subsequently may activate pro-inflammatory cytokine production through macrophages and dendritic cells [306–308]. Consequently, several studies in vivo and in vitro showed that vitamin D supplementation inhibits the Th2 expression contrasting allergic diseases [309–314]. Furthermore, previous studies focused on the association between serum 25(OH)D levels and the expression of genes involved in the proliferation and cells differentiation, showing an anti-proliferative activity of vitamin D with consequently remodeling inhibition [309–312]. Experimental data suggests that vitamin D_3_ significantly overcame the inhibition of glucocorticoid-receptor expression by dexamethasone while IL-10 upregulated glucocorticoid-receptor expression by CD4+ T cells, suggesting potential mechanisms whereby these treatments may overcome poor glucocorticoid responsiveness [315]. About the role of vitamin D in the immunopathogenesis of allergic skin diseases, vitamin D induces the production of antimicrobial peptides, as beta-defensin and cathelicidin, causing a reduction of the bacterial infections that may exacerbate asthma and atopic dermatitis (AD) [316, 317]. Not yet fully understood is the role of vitamin D on eosinophilic airway inflammation. In a recent study low 25(OH)D levels were associated to high levels of fractional exhaled nitric oxide [318]. On the contrary, in a study on 3130 mother child pairs there was no association between 25(OH)D levels and fractional exhaled nitric oxide in the first month of life [319].

Several studies evaluated the incidence of asthma in children and its relationship with 25(OH)D levels during pregnancy [320–323] and at delivery [252, 324]. Observational studies on the relationship between vitamin D in pregnancy and wheezing, asthma, and allergies development in early life showed unclear results. Recently, two studies on 581 and 806 children respectively showed that 2800 IU/day or 4400 IU/day of vitamin D_3_ administered during the third trimester of pregnancy compared to 400 IU/day did not show any statistically significant reduction on the risk of persistent wheezing in the offspring until 3 years of age [325, 326]. These results were confirmed in other studies that did not find without any correlation between serum 25(OH)D levels during pregnancy or at delivery and asthma development [252, 320–322, 324]. On the other hand, the study of Bener et al. in 966 children demonstrated that vitamin D deficiency [serum 25(OH)D <  20 ng/ml] was the major predictive factor of asthma risk [327], and Gale et al. showed that gestational 25(OH)D levels > 30 ng/ml were associated with an increased risk of asthma in children at 9 years of age [323].

Three studies evaluated 25(OH)D levels related to severity of asthma assessed by Asthma Control Test [328–330]. The first two studies showed that 25(OH)D levels were inversely related to asthma severity, while Gergen et al. demonstrated a correlation only for Afro-Americans subjects.

Regarding asthma exacerbations, several studies evaluated the association between 25(OH)D levels and the hospitalization for asthma and/or asthma treatment with oral steroids [330–333]. In most studies, low 25(OH)D levels were associated with a rise in hospital admissions or oral steroids treatment. A meta-analysis showed a significant association between vitamin D supplementation and reduction of asthma exacerbations (17% vs. 46%, *p* < 0.029) [334].

Recently, three studies showed a positive correlation between vitamin D supplementation and the improvement of asthma control [335–337], accordingly with two recent meta-analyses. Vitamin D supplementation regimen changed in the studies between 500 and 2000 IU/day [338, 339]. Only the study of Lewis et al. did not show any correlation between 25(OH)D levels and asthma control [340].

About the effects of vitamin D on lung function, a recent meta-analysis demonstrated a difference of 0.54 l/s in forced expiratory volume in the 1st second (FEV1) values after the treatment with vitamin D [334]. In another study high doses of vitamin D (100,000 IU as first dose and 50,000 IU/week for 6 months) were administered to 130 patients (children and adults). After 28 weeks the authors showed an increase (20%) in FEV1 values in the patients treated with vitamin D and corticosteroids in comparison with an increase of 7% in the control group [341]. Vitamin D supplementation at 2000 IU/day for 6 weeks in 39 children with mild asthma and serum 25(OH)D levels < 30 ng/m) did not showed significant variations in bronchial reactivity or inflammatory markers (IL-4, IL-5, IL-10, IL-17, and interferon-gamma) [342]. Two studies in vitro showed the importance of vitamin D supplementation in patients affected by corticosteroid-resistant asthma [315, 343] but two other studies demonstrated in vivo that lower 25(OH)D levels were associated with an increase in corticosteroids use and an impaired lung function [344, 345].

### Atopic dermatitis and allergic diseases

Atopic dermatitis (AD) is a common chronic disease with symptoms starting from the early childhood. It has been observed in several studies that in moderate-severe AD serum 25(OH)D levels are often reduced [346]. Being vitamin D involved in innate immunity and in skin barrier integrity, and because both factors are dysfunctional in AD, the association is actively debated. The evidences for a pathogenetic role for vitamin D in AD are still conflicting even though data in favour of such correlation are increasingly stronger with more studies in favour but others opposing this hypothesis. However, at least two longitudinal studies have correlated the vitamin D cord blood low levels to the development of AD in the offsprings, finding that low levels in the cord blood significantly increased the risk to develop the disease [347, 348]. The lower intake of vitamin D during early childhood increased the risk for disease persistence during the mild childhood [348].

It is still a matter for full debate whether supplementation with vitamin D in pediatric AD is worth, particularly in deficient patients. A recent large study found no evidence that genetically determined reduction in 25(OH)D levels conferred an increased risk for AD, suggesting that efforts to increase vitamin D are unlikely to reduce risks of the atopic disease [349]. However, there are also two reviews and meta-analyses, considering AD patients of all ages, showing that vitamin D supplementation is capable of a higher mean difference in severity of AD symptoms [350, 351]. Particularly, vitamin D supplementation decreased AD severity and improved its symptoms and clinical signs. In pediatric population there is some evidence about a favourable effect of administration of vitamin D on several aspects of AD, but results are still conflicting. For these reasons at present vitamin D supplementation as adjunctive standard therapy for AD cannot be recommended in all the affected children [351]. There is a need for RCTs conducted on broader populations, with a prolonged follow up. However, it has been suggested that in patients with a more severe AD, unresponsive to common therapy and with low serum 25(OH)D levels, a trial of vitamin D supplementation can be considered [352]. This could be particularly indicated during the winter season where the sun exposure is limited and ineffective and supplementation not already suggested. Again, we need clinical data showing that restoring normal levels of vitamin D one can obtain significant variation in severity especially if evaluated by standardized methods (symptom score, Scoring Atopic Dermatitis or equivalent).

The so-called ecological studies suggest the relationship between latitude, the consequent level of sun exposure, vitamin D production and subsequent serum 25(OH)D levels and the prevalence and severity of allergic diseases. However, data from observational studies even though encouraging, are still controversial [353]. Furthermore, the use of vitamin D supplementation for primary prevention of allergic diseases remains an attractive area of study, but current knowledge and evidence does not allow to recommend it in routine use. Data from ongoing intervention trials are warranted to establish the extent of vitamin D potentially preventive effect, along with the best timing and dose of an intervention.

Also in food allergies the role of vitamin D remains controversial. There are studies suggesting that both lower and higher levels of vitamin D are associated with elevated IgE concentrations, with a U-shaped relationship [354]. However, evidences are still subtle and conflicting. A recent systematic review failed to demonstrate a significant association between serum 25(OH)D levels and food allergies prevalence [355]. Negative results were obtained in term of primary prevention by vitamin D supplementation in a recent meta-analysis and conclusions were that the effects of vitamin D supplementation remain uncertain [353]. There is the need for RCTs to validate these hypotheses and to evaluate the possibility of primary prevention and treatment of food allergies with vitamin D [355–357].

### Type 1 diabetes mellitus

Several epidemiological studies suggested that type 1 diabetes mellitus (T1DM) is associated with vitamin D deficiency. A north-south gradient in the incidence of T1DM as well as a seasonal pattern of disease onset have been described. Low serum 25(OH)D levels are associated to T1DM in children and adolescents independently by ethnic origins and environmental living conditions (latitude, altitude) [358], persisting over time [359–366]. Moreover, 25(OH)D levels were lower in some but not all studies on siblings at risk [367, 368]. Some studies suggest a linkage between polymorphisms of the VDR and T1DM with data more convincing in Asians [369–371]. A recent large meta-analysis focused only on pediatric populations revealed that *Bsm*IBB, *Bsm*IBb and *Taq*Itt polymorphisms were associated with an increased risk of T1DM, whereas *Bsm*Ibb and *Taq*ITT had protective effect [372]. Data on polymorphisms of other genes involved in vitamin D metabolism are still conflicting or related to small populations [373–376].

A meta-analysis of six case-control studies and two cohort-studies showed a reduction of the risk of T1DM in later life (odds ratio: 0.71; 95% CI 0.51–0.98) in infants who were supplemented with vitamin D compared with controls, although the needed time of supplementation and the dose are still unclear [377–379]. On the other hand, data on supplementation in pregnancy are debated, and no evidence of protection has been shown by recent meta-analyses [379, 380].

If data of observational studies in general population in infancy are encouraging, in the DAISY study, conducted in subjects at higher risk, the vitamin D nutritional intake in the first years of life as well as the serum 25(OH)D levels at 9 months of age were not associated with the development of autoantibodies or with the progression to T1DM [381]. Conversely, neonatal vitamin D status seems to be associated to the risk of T1DM in some [382] but not all the populations [383]. Moreover, 25(OH)D levels were not associated with a fast progression to T1DM later in life [368].

Until now, few clinical studies using cholecalciferol, calcidiol, calcitriol or its analogs have been conducted in the prevention or T1DM or in children with a recent onset. They are small, short-term and often not randomized. The IMDIAB XI study in which new onset T1DM children were given in the vitamin D group calcitriol at 0.25 μg/2 days or nicotinamide at 25 mg/kg/day reported a reduction of insulin requirements in the vitamin D group [384]. However, the IMDIAB XIII study in which adolescents and young adults were given calcitriol 0.25 μg/day vs. placebo for 2 years failed to demonstrate any improvement in T1DM [385]. Higher doses of cholecalciferol (2000 IU/day, 4000 IU/day, 70 IU/Kg/day, or 14000 IU/month) improved the immunity status in small populations with T1DM [386–389]. Similar data were obtained with calcidiol for 1 year or calcitriol for 6 months in two pilot studies [390, 391].

Although some suggestive and encouraging data on epidemiological observational studies and pilot clinical studies of intervention, the lack of evidence deriving by RCTs does not allow to encourage treatment with cholecalciferol or other vitamin D metabolites in patients with T1DM or subjects at higher risk. However, vitamin D deficiency should be avoided in these populations.

### Inflammatory bowel diseases

Vitamin D status has been associated with the pathogenesis of several autoimmune diseases, including inflammatory bowel disease (IBD) [392–395]. Interestingly, the incidence of pediatric Crohn’s disease increases with higher latitude and greater number of months with low ambient ultraviolet radiation [396], and some studies showed high prevalence of hypovitaminosis D in IBDs children and adolescents [397, 398]. A recent meta-analysis showed that patients with Crohn’s disease (*n* = 129808, age 11–48 years) had lower serum 25(OH)D concentrations compared with healthy controls, with an overall prevalence of vitamin D deficiency [25(OH)D <  20 ng/ml] of 57.7% and an inverse correlation between serum 25(OH)D levels and the severity of disease [399]. The etiology of vitamin D deficiency in patients with IBD is multifactorial and not entirely known. Besides risk factors common to general population (seasonality, reduced sun exposure, low dietary vitamin D intake, etc.) subjects with IBD had increased risk due to malabsorption, intestinal inflammation, protein-losing enteropathy, and steroid treatment. Patients at early stage of IBD, with more severe disease, and upper gastrointestinal tract involvement are at higher risk for deficiency [398, 400–402].

Clinical guideline for skeletal health of children and adolescents with IBD in 2011 recommended monitoring vitamin D levels at least yearly, at the end of winter/beginning of spring, especially in dark skinned subjects. The authors suggested particular attention in children with active IBD, low albumin level (< 3 g/dl), and evidence of nutritional impairment [403]. The North American Society for Pediatric Gastroenterology, Hepatology and Nutrition [404] and other authors [405, 406] also recommended to evaluate serum 25(OH)D levels in IBD children at diagnosis and at least yearly, preferably in the spring. A dietary assessment should also be routinely performed in IBD children, paying close attention to the consumption of vitamin D-containing foods and dairy products [404].

At present the ideal dosing regimen to treat vitamin D deficiency and to maintain sufficiency in IBD patients is still debated [406]. In IBD pediatric patients, 2000 IU/day of vitamin D_3_ for 6 months was more effective in raising serum 25(OH)D concentrations > 30 ng/ml than 400 UI/day or doses up to 2000 IU of vitamin D_2_ [407, 408]. On the other hand, supplementation with 2000 IU/day of vitamin D_3_ for 13.8 months improved trabecular BMD, cortical bone cross-sectional area, and maximal muscle power in 55 IBD patients [409].

Regarding dose regimens, both oral doses of 2000 IU/day of vitamin D_3_ and 50000 IU/week of vitamin D_2_ for 6 weeks were well-tolerated and superior to 2000 IU/day of vitamin D_2_ in raising serum 25(OH)D concentration IBD patients with vitamin D deficiency [410]. Simek et al. found that two regimens of weekly vitamin D_3_ administration (5000 IU/10 Kg/week, maximum weekly dose of 25000 IU and maximum cumulative dose of 150000 IU vs. 10000 IU/10 Kg/week, maximum weekly dose of 50000 IU and maximum cumulative dose of 300000 IU) for 6 weeks were safe and effective in normalizing vitamin D status in IBD children and adolescents with hypovitaminosis D [411]. Finally, a single age dependent high-dose oral vitamin D_3_ (the so-called stoss therapy; < 3 years: 200000 IU; 4–12 years 400000 IU; > 12 years 800000 IU) was safe and efficacious in achieving and maintaining serum 25(OH)D levels > 20 ng/ml during a 6-month period in IBD children with vitamin D deficiency [412].

Clinical guideline for skeletal health of children and adolescents with IBD recommend to treat vitamin D deficiency and insufficiency administering a cumulative dose of at least 400000 IU, and 250000 IU of vitamin D, respectively. Vitamin D supplementation at 800–1000 IU/day is suggested to maintain optimal vitamin D status [403]. The Endocrine Society recommends to treat vitamin D deficiency in patients with malabsorption with vitamin D at doses 2–3 times higher than those recommended for otherwise healthy pediatric population for at least 6 weeks, followed by vitamin D supplementation at doses 2–3 times higher than those recommended for age [11]. Recently, Ahlawat et al. suggested to treat pediatric IBD subjects with hypovitaminosis D at least with 2000 IU/day of vitamin D_3_, adjusting every 3 months depending on serum 25(OH)D levels [406].

The role of vitamin D as a therapeutic agent in IBD is still under investigation. Particularly, large RCTs are needed to better investigate the role of vitamin D in disease remission and control.

### Celiac disease

Celiac disease (CD) is caused by dysregulated systemic and intestinal mucosal immune responses to dietary gluten proteins in genetically predisposed individuals. It has been suggested that early-life vitamin D deficiency may contribute to the pathogenesis of childhood-onset CD by determining inappropriate immune responses, abnormal intestinal mucosal integrity and impaired local defence against microbial agents [413]. A relationship between sun exposure and CD pathogenesis has been suggested demonstrating that CD was more common in US individuals living at northern latitudes than at southern latitudes (odds ratio 35°-39°N: 3.2; odds ratio ≥ 40°N: 5.4 vs. < 35°N) [414]. On the contrary, maternal vitamin D supplementation, maternal and neonatal vitamin D status were not related to the risk of childhood CD [415, 416].

Vitamin D deficiency is common (up to 52%) in children with CD at diagnosis [417–421]. Untreated CD is independently associated with reduced BMD in children and adolescents, as confirmed by US data from the National Health and Nutrition Examination Survey 2009–2010 and 2013–2014 [422], with increased risk of osteoporosis and fragility fractures in adulthood [423]. Indeed, small intestinal mucosal damage may affect intestinal absorption of calcium and vitamin D, and chronic intestinal inflammation may lead to release of proinflammatory cytokines with subsequent increased bone loss [424]. Strict adherence to gluten free diet results in recovery of BMD in both children and adults [417, 425, 426], and the promotion of an adequate vitamin D status is important to optimize bone health in CD patients [419].

The American College of Gastroenterology [427] and the British Society of Gastroenterology [428] recommend serum 25(OH)D evaluation in adults with CD at the time of initial diagnosis. The North American Society for Pediatric Gastroenterology, Hepatology and Nutrition in 2016 recommend serum 25(OH)D evaluation also in CD children at diagnosis and annually after symptoms resolution and normalization of CD serology [429]. Recent evidence-informed expert recommendations for the management of CD in children suggest to evaluate serum 25(OH)D levels at diagnosis, while follow-up evaluation is needed only if previously abnormal (grade of evidence low, strength of statement weak) [430]. Other authors gave similar recommendations in a recent review [406].

Spontaneous recovery of vitamin D status following gluten free diet has been suggested [417], but it is still debated. Strict adherence to gluten free diet itself may represents a determinant of vitamin D status, because it has been demonstrated that Sweden CD children on gluten free diet had low vitamin D intake compared with Nordic Nutrition Recommendation and lower nutrient density of vitamin D than healthy children [431].

As vitamin D is an important key determinant of bone mass acquisition, some authors recommended vitamin D supplementation during winter and spring in all CD children and adolescents to optimize bone recovery [432]. NICE guidelines advice that people with CD may need to receive calcium and vitamin D supplementation if their dietary intake is insufficient [433]. Other authors reinforce that vitamin D supplementation should be offered to CD patients at least during the first years of gluten free diet [423]. Expert recommendations for the management of CD in children recommended to provide counselling on age-appropriate intake of calcium and vitamin D supplementation by a dietitian to CD children both at diagnosis and follow-up (grade of evidence high, strength of statement strong) [430]. Future research will clarify the ideal vitamin D dosing regimen to treat vitamin D deficiency and to maintain serum 25(OH)D levels ≥30 ng/ml in CD children and adolescents [406].

### Obesity and metabolic syndrome

Vitamin D status is influenced by adiposity because adipose tissue represents a site of storage for lipophilic substances [434, 435]. In fact, obese children frequently show reduced serum 25(OH)D levels [66, 436]. Particularly, obese Italian children and adolescents have high prevalence of vitamin D deficiency and insufficiency [50, 71, 75, 78–80, 83, 84, 437, 438]. Two meta-analyses confirmed that vitamin D deficiency was associated with obesity in children, adolescents and adults irrespective of age, latitude, and cut-offs used to define deficiency [439, 440].

Conversely, vitamin D deficiency does not lead to obesity [441]. A recent meta-analysis of 4 RCTs and 11 non-RCTs, only 1 enrolling children [442], showed that weight loss marginally improved vitamin D in comparison with weight maintenance under similar vitamin D intake [443], confirming that increased adiposity causes suboptimal serum 25(OH)D concentrations. On the contrary, two meta-analyses enrolling mainly adults showed that vitamin D supplementation did not decrease adiposity [444, 445]. Animal models and in vitro studies report that vitamin D may influence both pancreatic insulin secretion and insulin sensitivity in skeletal muscle [446, 447]. Thus, several authors have suggested that vitamin D deficiency could play a role in worsening the comorbidities of pediatric obesity. In 2009, Reis et al. reported that obese children with lower serum 25(OH)D levels had a higher risk for low high density lipoprotein cholesterol, high blood pressure, and elevated fasting glucose [448]. Few years later, Roth et al. showed an inverse relationship between serum 25(OH)D levels and insulin resistance [449]. In 2007, Reinehr et al. reported no correlation between insulin sensitivity and vitamin D levels, even after weight loss [442]. Moreover, Erdönmez et al. showed no difference in vitamin D status between obese with or without impaired glucose homeostasis [450]. Further, contrasting results came from the studies investigating the association between vitamin D deficiency and non-alcoholic fatty liver disease [451–453].

Several RCTs have been conducted to evaluate the efficacy of vitamin D supplementation as a potential therapy for each component of metabolic syndrome. In a 16-week RCT with 2000 IU/day of vitamin D authors reported that therapy was able to reduce arterial stiffness in black obese adolescents [151]. Other papers describe a significant reduction of insulin resistance index after vitamin D supplementation [454, 455]. A prospective study showed that high dose vitamin D_2_ treatment (20000 IU/day for 28 days) increased insulin sensitivity and improved abnormal glucose metabolism in obese children [456]. More recent findings have reported no influence of different vitamin D supplementation schedules on serum lipid levels, glucose homeostasis, glycated hemoglobin and pancreatic insulin secretion [457–459]. A recent Italian RCT showed that supplementation with vitamin D (800 IU/day) plus docosahexanoic acid improved insulin-resistance, lipid profile, aminotransferases levels, and activity score in obese children with non alcoholic fatty liver disease and vitamin D deficiency [460]. Finally, two recent meta-analyses showed that vitamin D supplementation had no significant effect on glucose and insulin metabolism [461] and on changes in the concentrations of inflammatory biomarkers (C-reactive protein, tumor necrosis factor alpha, IL-6) in obese and overweight adolescents and adults [462].

At present the need for evaluate serum 25(OH)D levels in obese children is still debated. The Endocrine Society [11], United Arab Emirates guidelines [27] and The Society for Adolescent Health and Medicine [21] recommended to screen all obese children and adolescents as they are at increased risk for vitamin D deficiency. On the contrary, the AAP suggested that more evidence is needed before recommendations can be made regarding screening of obese subjects [23].

Different vitamin D dose regimens have been proposed in obese children and adolescents for both vitamin D supplementation and treatment of deficiency [463–467]. The Endocrine Society Clinical Practice Guideline suggested in obese children vitamin D supplementation at doses 2–3 times higher than those recommended for age to satisfy their body’s vitamin D requirement. Similarly, obese children with vitamin D deficiency should be treated with vitamin D at doses 2–3 times higher than those recommended for non-obese pediatric population for at least 6 weeks [11]. Similarly, Guidelines for Central Europe recommended in obese children vitamin D supplementation (1200–2000 IU/day depending on severity of obesity) between September and April, or throughout the whole year if sufficient skin synthesis of vitamin D is not ensured in the summer [20]. Regarding adolescence, the Society for Adolescent Health and Medicine recommended that obese adolescent without a 25(OH)D measurement empirically receive vitamin D supplementation at 1000 IU/day [21]. More recently, United Arab Emirates guidelines also recommended vitamin D supplementation in obese children and adolescents (1200–2000 IU/day depending on severity of obesity, season and sun exposure) throughout the year if sufficient skin synthesis if vitamin D is not ensured during winter [27]. Differently, SACN confirmed that obese people are at risk of low serum 25(OH)D concentrations, but highlighted that currently evidence is insufficient to recommend for obese individuals different reference nutrient intake of vitamin D from that proposed for the general UK population [25]. Recently, a double-blind RCT analyzing the effects of vitamin D_3_ supplementation (1200 IU/day for 26 weeks vs. placebo) in overweight or obese children with hypovitaminosis D has been proposed [468].

In conclusion, obese children have higher risk of vitamin D deficiency and insufficiency. It is still unknown the role of this nutrient in the natural history of obesity and its comorbidities. More RCTs are needed to evaluate the influence of vitamin D supplementation on controlling metabolic syndrome. Standardization of study design and supplementation dose should improve the evaluation of the efficacy of supplementation. At present, considering that obese Italian children have higher prevalence of vitamin D deficiency and insufficiency then normal weight individuals, we suggest in obese children and adolescents vitamin D supplementation at higher doses than those recommended for age (1000–1500 IU/day) from the end of fall to the beginning of spring (November–April) to ensure an adequate vitamin D status. Obese subjects with reduced sun exposure during summer should receive vitamin D supplementation throughout the year. Finally, sensible sunlight exposure and outdoor physical exercise should be encouraged in obese children and adolescents.

### Autism and depression

Autism is a complex disease that compromises nonverbal communication. Several observational studies have linked autism spectrum disorders and low serum 25(OH)D levels during pregnancy because of vitamin D pleiotropic activity [469–476]. Moreover, autism spectrum disorders affect dietary habits leading to food selectivity, thus patients could develop multiple nutritional deficiencies [477–479]. Particularly, a recent meta-analysis showed that serum 25(OH)D levels in children and adolescents (age 2–16 years) with autism spectrum disorders were significantly lower than controls, suggesting that lower vitamin D levels might be a risk factor for autism [480]. Few studies or case reports suggested that vitamin D supplementation can improve autism symptoms [481–484]. Nevertheless, a recent RCT enrolled 42 children with autism spectrum disorders showing that vitamin D supplementation (2000 IU/day of vitamin D_3_ for 20 weeks) had no effect on the primary outcome (the stereotypic behaviour subscale from the Aberrant Behaviour Checklist) in comparison with placebo [485]. Therefore, further RCTs are needed before recommending routine vitamin D supplementation in children with autism.

Depression affects about 1–6% of children [486]. Vitamin D deficiency has been proposed as a possible cause of depression. In fact, VDR is expressed in different brain’s areas involved in the pathogenesis of the disease [487, 488]. Moreover, the severity of symptoms is higher in winter when sun exposure is low [489]. Many studies report an association between inadequate serum 25(OH)D levels and a higher rate of depression or severity of symptoms both in adults and children [490–492]. In contrast, Fazeli et al. found no differences in vitamin D status and bone density among adolescents affected by major depression and healthy controls [493]. In agreement, Tolppanen and coworkers reported no association between serum 25(OH)D levels and any mood disorders [494]. A small Swedish study showed that depressed adolescents with serum 25(OH)D levels < 24 ng/ml supplemented with vitamin D3 (4000 IU/day for 1 month followed by 2000 IU/day for 2 months) improved symptoms related to depression [491]. Meta-analyses assessing the effect of vitamin D supplementation in depressed adults founded conflicting results [495–497]. As literature shows contrasting results because of different methods and study designs, more studies are needed to define the role of vitamin D in pathogenesis and severity of depression, particularly in the pediatric age.

## Pregnancy and breastfeeding

The high prevalence of vitamin D deficiency in pregnant women is a worldwide health problem regardless of latitude, food intake or socio-economic status [93]. Data about vitamin D intake and status in pregnant women are relatively scarce but confirm this scenario. A recent Italian study [81] evaluated serum 25(OH)D levels at term of pregnancy in Italian and immigrant women and in cord blood: 18% and 43.6% of Italian, 48.8% and 41% of immigrant women had severe (< 10 ng/ml) or mild (< 20 ng/ml) 25(OH)D deficiency, respectively. Likewise, severe 25(OH)D deficiency was found in 38% of Italian and in 76.2% of foreign newborns. Semi-quantitative food questionnaires showed that the average dietary vitamin D intake is 136 ± 68 IU, significantly lower than the Institute of Medicine recommended amount (600 IU) [10]. Another Italian study [498] examined 24 women (light and dark skinned) at full-term pregnancy: 23 out of 24 women had vitamin D deficiency. Similarly, both light and dark skinned babies showed vitamin D deficiency at birth.

During pregnancy vitamin D metabolism changes so that maternal serum 1,25(OH)_2_D levels significantly rise to meet increased calcium requirements for fetal skeleton mineralization, whereas fetal vitamin D stores are exclusively dependent on maternal vitamin D status. The increase of 1,25(OH)_2_D in the mother plays a key role in modulating calcium homeostasis in both mother and fetus, first of all doubling maternal intestinal calcium absorption [499]. Moreover, maternal bone turnover is reduced in the first half of pregnancy, followed by the progressive increase of bone resorption, reaching the maximum in the 3rd trimester of pregnancy, to ensure fetal supply of calcium for fetal bone development. In addition, vitamin D plays a key role in the immunological adaptation needed for the onset and maintenance of a normal pregnancy [500].

Increasing scientific evidence suggests that hypovitaminosis D in the mother may have an impact on both maternal and child health. Reduced 25(OH)D levels during pregnancy and breastfeeding may affect maternal bone turnover [501]. Moreover, maternal vitamin D deficiency has been associated with increased risk of several obstetrics diseases (spontaneous pregnancy loss, preterm and/or small-for-gestational age birth, gestational diabetes pre-eclampsia) [502–506]. In addition, vitamin D deficiency during pregnancy may negatively affect long-term offspring extraskeletal health outcomes, with increased risk of infant respiratory tract infections, asthma, wheeze, and eczema [273, 380, 507, 508].

Vitamin D supplementation during pregnancy can improve maternal and cord blood 25(OH)D concentrations in women with low 25(OH)D levels [509]. Supplementing pregnant women with vitamin D in a single or continued dose increases serum 25(OH)D at term and may reduce the risk of preeclampsia, low birth weight and preterm birth. However, larger and better-designed controlled randomized trials are required to confirm these effects [510].

The Royal College of Obstetricians & Gynaecoloists recommended vitamin D supplementation with 400 IU/day for all pregnant women. Higher doses (at least 1000 IU/day) may be required in high-risk women (women with increased skin pigmentation, reduced exposure to sunlight, or those who are socially excluded or obese) [511]. Indeed, vitamin D supplementation with 400 IU/day has been reported insufficient in pregnancy, particularly for women at higher risk of vitamin D deficiency [512, 513]. More recently, Wagner et al. suggested that a vitamin D intake of at least 4000 IU/day should be considered in all pregnant women to maintain serum 25(OH)D levels ≥40 ng/ml [513, 514].

Screening for vitamin D deficiency during pregnancy is still debated. For example, the Endocrine Society considered gestation as a period at significant risk for deficiency and thus recommended serum 25(OH)D evaluation in all pregnant women [11]. More recently, the Royal College of Obstetricians & Gynaecoloists suggested to evaluate 25(OH)D concentrations during pregnancy in selected case (Table 9) [511]. Moreover, the Society for Adolescent Health Medicine recommended routine 25(OH)D testing in pregnant adolescents [21].

**Table 9:** Indications for serum 25(OH)D evaluation during pregnancy (modified from [511])

• Non-Caucasian ethnicity with dark skin pigmentation	
• Reduced sunlight exposure (i.e. veiled women) and/or constant use of sunscreens	
• Malabsorption syndromes	
• Chronic therapies affecting vitamin D metabolism	
• Obesity	
• Risk of pre-eclampsia	
• Alcohol abuse	
• Bone pain	
• Hypocalcemia	
• A previous child with rickets	
• Pregnant adolescents	

In the present Consensus we recommend vitamin D supplementation in all pregnant and breastfeeding women since the beginning of pregnancy at a dose of 600 IU/day. Women with risk factors for vitamin D deficiency should receive higher dosages (1000–2000 IU/day). We do not recommend routine screening of serum 25(OH)D levels in all pregnant and breastfeeding women, but we suggest to consider vitamin D testing in women with multiple risk factors for vitamin D deficiency, particularly if not receiving vitamin D supplementation.

## Appendix. Management of asymptomatic vitamin D deficiency and insufficiency

In recent years some international Societies and Committees proposed various regimens to treat of vitamin D deficiency [9, 11, 20–23]. The AAP recommended to treat vitamin D deficiency administering 2000 IU/day or 50,000 IU/week of vitamin D_2_ or D_3_ (independent of weight) for 6 week in infants (0–12 months) and for 6–8 weeks in children and adolescents (1–18 years), suggesting that vitamin D_3_ may be more potent than vitamin D_2_ [23]. After completion of treatment, serum 25(OH)D concentrations should be revaluated, as it is not unusual for a second course of treatment to be necessary to achieve adequate vitamin D status [23]. Particularly, maintenance therapy with standard doses or with doses two or three times higher than those recommended for healthy children is recommended after treatment [9, 11, 20–23].

Relatively few studies evaluated the efficacy of different regimens with vitamin D_2_ or vitamin D_3_ for the treatment of vitamin D deficiency in otherwise healthy children.

### Studies with daily or weekly regimens

In vitamin D deficient infants younger than 2 years, either 2000 IU of vitamin D_2_ or vitamin D_3_ daily or 50000 IU of vitamin D_2_ weekly for 6 weeks equally increased serum 25(OH)D levels [515]. A recent RCT in children (2–5 years) with vitamin D deficiency showed that treatment with oral 4000 IU/day or 30000 IU/week for 12 weeks or a single intramuscular dose of 300000 IU of vitamin D_3_ followed by a maintenance dose of 400 IU/day increased and maintained serum 25(OH)D concentration > 30 ng/ml after 1 year [516]. Daily administration of 2000 IU of vitamin D_3_ for 16 weeks was effective in increasing serum 25(OH)D levels > 30 ng/ml also in black adolescents (14–18 years) with vitamin D deficiency [151]. Another RCT showed that adult-size adolescents require high-dose vitamin D_3_ (at least 5000 IU/day for 8 week) to correct deficiency [517]. Particularly, as discussed before, the administration of 50000 IU/week for 6–8 weeks may be insufficient to increase 25(OH)D concentration > 30 ng/ml in obese children and adolescents [466, 517]. The administration of 60000 IU of vitamin D_3_ weekly for 4–8 weeks followed by 600 IU daily for 12 weeks in Indian adolescents [518] or a regimen of 60000 IU/week of vitamin D_3_ for 8 weeks followed by 60000 IU/fortnight in young Indian females [519] were both effective in increasing and maintaining 25(OH)D levels in deficient individuals. Adolescents treated with 14000 IU of vitamin D_3_ weekly (equivalent to 2000 IU daily) for 1 year normalized their 25(OH)D levels [166, 167, 520]. A recent 1-year-RCT compared three different regimens for asymptomatic children and adolescents with vitamin D deficiency and insufficiency (400 IU/day, 45000 IU weekly for 2 months then 400 IU daily, 2000 IU/day for 3 months then 1000 IU/day), suggesting that low loading dose with high maintenance dose is preferred to achieve steady increase in serum 25(OH)D levels avoiding hypercalcemic side effects [521]. Lower doses as 200 IU/day, 400 IU/day, 800 IU/day [147, 153] or 1400 IU/week for 1 year [166], 1000 IU/day for 6 months [159], or 5000 IU/week for 8 weeks [165] were not able to normalize 25(OH)D levels in several pediatric populations, including Italian children and adolescents supplemented with 400 IU/day for 1 year [78].

### Studies with monthly or other regimens

The majority of these studies have been conducted in small populations of adolescents. Several schedules have been used, with conflicting results.

The administration of 60000 IU/month of vitamin D_3_ for 12 months in Indian children was effective in raising 25(OH)D concentrations > 30 ng/ml [171], while another study showed that 60000 IU of vitamin D_3_ monthly or every 2 months for 12 months in Indian females (6–17 years) increased 25(OH)D levels in the range of insufficiency [172]. Similarly, a RCT in Iranian adolescents showed that 50000 IU/months of vitamin D_3_ for 5 months increased serum 25(OH)D levels but remained insufficient [170]. Two studies evaluated the efficacy of administering a bolus dose of vitamin D_2_, 150000 IU [225] or 300000 IU [224], every 3 months for 12 months in adolescent females with severe vitamin D deficiency, with conflicting results. Indeed, only in the study of Khadilkar et al. serum 25(OH)D concentrations increased > 30 ng/ml. Adolescents with severe vitamin D deficiency treated with an intramuscular injection of a megadose of vitamin D_3_ (10000 IU/Kg, maximum 600000 IU) had insufficient serum 25(OH)D after 3 months [522]. Both 300000 and 600000 IU single oral vitamin-D bolus were effecting in treating vitamin D deficiency in young children (3 months-3 years) but a significant percentage developed hypercalcemia and/or hypercalciuria [195]. On the contrary a stoss therapy (oral single-dose of 10000 IU/Kg or 300000 IU of vitamin D_3_) has been demonstrated effective and safe in the treatment of vitamin D deficiency in older children (mean age 10.6 ± 4.4 years) without rickets [523]. However, at present safety data in both the short- and the long-term of monthly or other vitamin D regimens lack due to the nature of the studies, especially in young children.

Different dose regimens have been proposed in conditions and diseases known to be a risk for vitamin D deficiency. However, all the studies have been conducted in small populations.

Regarding children receiving anti-retroviral drugs, Kakalia et al. found that in children infected with HIV and vitamin D insufficiency a daily intake ≥1600 IU/day of vitamin D_3_ vitamin D may be required to achieve serum 25(OH)D levels > 30 ng/ml [524]. Higher doses of 4000 or 7000 IU/day of vitamin D_3_ for 6–12 weeks or 1 year increased 25(OH)D levels in the range of sufficiency in the majority of the patients with a variable prevalence of hypercalcemia which required no clinical intervention [289, 525–527]. Differently, regimens with 50,000 IU monthly [528] or 100000 IU of vitamin D_3_ every 2–3 months normalized 25(OH)D levels only in a half of the patients [288, 529, 530]. In children on long-term antiepileptic drugs (valproate, lamotrigine, topiramate, clonazepam, gabapentin, carbamazepine, phenytoin, phenobarbital), the administration of 2000 IU/day of vitamin D_2_ for 1 year slightly increased mean 25(OH)D level from 18.0 ± 9.1 to 22.9 ± 8.4 ng/ml [531]. No trials with different vitamin D_2_ or vitamin D_3_ regimens have been conducted in children under chronic corticosteroid treatments. For treatment of vitamin D deficiency in obese or IBD children see specific chapters.

At present there is no strong evidence about whether to treat asymptomatic children with vitamin D insufficiency [23]. The Society for Adolescent Health and Medicine recommended to supplement vitamin D insufficient with vitamin D 1000 IU/day for at least 3 months [21]. In the case of vitamin D insufficiency, particularly in subjects at risk for vitamin D deficiency, we recommended starting vitamin D supplementation according to the modalities and requirements recommended for age.

## Conclusions

Vitamin D status is a key determinant of bone health during childhood and adolescence. Nutritional rickets cases are still reported in Italy, thus adequate national strategies to prevent vitamin D deficiency are needed. Moreover, the recently suggested role of vitamin D in the development of other non-skeletal diseases reinforced the interest in the promotion of an adequate vitamin D status during pediatric age. The present Consensus paper aims to give practical approach to vitamin D supplementation for Italian infants, children and adolescents. Particularly, vitamin D supplementation should be recommended in all infants in the first year of life, independently of the type of feeding. Supplementation should be subsequently individualized in terms of regimen and duration on the basis of the presence of risk factors for vitamin D deficiency. More studies, particularly RCTs, are needed to confirm the promising role of vitamin D in the promotion of the global health of children.

## Summary of recommendations

### Definition of vitamin D status

- Individual vitamin D status can be assessed evaluating serum circulating 25(OH)D levels.
- Depending on 25(OH)D levels, vitamin D status can be defined as follows:
  - Sufficiency ≥30 ng/ml
  - Insufficiency 20–29 ng/ml
  - Deficiency < 20 ng/ml
  - Severe deficiency < 10 ng/ml
- The term hypovitaminosis D refers to serum 25(OH)D levels < 30 ng/ml.

### Evaluation of vitamin D status

- The isotope dilution- LC-MS/MS is considered the preferred method for measuring serum 25(OH)D levels, especially in the neonatal period. However, considering the reduced availability on the Italian territory of this method, other reliable immunoassay methods can be used if performed in certified laboratories, with the exclusion of neonates.

### Prevalence of hypovitaminosis D in Italy in pediatric age

- Available epidemiological studies show a high prevalence of hypovitaminosis D (above 50%) throughout Italy. Adolescents are particularly at risk of hypovitaminosis D.
- Vitamin D status of newborns is influenced by ethnicity, season of birth, and maternal vitamin D status during pregnancy.
- Vitamin D status of children and adolescents is influenced by sun exposure, seasonality, ethnicity, and body mass index.

### Vitamin D supplementation

#### 0–12 months

- We recommend vitamin D supplementation in the first year of life to ensure an adequate vitamin D status and to prevent nutritional rickets.
- We recommend vitamin D supplementation in all newborns independently of the type of feeding.
- Vitamin D supplementation should be started within the first days of life and continued throughout the first year.
- Infants born at term without risk factors for vitamin D deficiency should receive 400 IU/day of vitamin D.
- In the presence of risk factors for vitamin D deficiency (Table 6) up to 1000 IU/day of vitamin D can be given.
- In the first year of life we recommend daily administration of vitamin D.
- We recommend against using vitamin D metabolites and their analogs (calcifediol, alfacalcidol, calcitriol, and dihydrotachysterol) for the routine vitamin D supplementation. The administration of these compounds increases the risk of hypercalcemia and is not able to maintain and/or restore vitamin D stores.
- We recommend against routine 25(OH)D testing in infants in the first year of life. We suggest to measure serum 25(OH)D levels in infants with multiple risk factors for vitamin D deficiency (Table 6).

#### Preterm infants

- We suggest for VLBW infants a vitamin D intake of 200–400 IU/day (including the amount administered through parenteral nutrition, fortified breast milk, or preterm infant formula).
- When VLBW infants reach a weight ≥ 1500 g and full enteral nutrition we suggest vitamin D supplementation at 400–800 IU/day.
- We recommend vitamin D supplementation at 400–800 IU/day for preterm infants with birth weight ≥ 1500 g.
- After a post-conceptional age of 40 weeks, recommendations for vitamin D supplementation are equal to those for healthy term infants.
- We recommend against routine 25(OH)D testing in preterm newborns.

#### 1–18 years

- We recommend vitamin D supplementation in children and adolescents with risk factors for vitamin D deficiency (Table 7). Moreover, we recommend to evaluate modifiable life-style risk factors for deficiency, particularly a reduced sun exposure. Ensuring an adequate vitamin D intake is particularly important during adolescence.
- We recommend daily vitamin D supplementation ranging from 600 IU/day (i.e. in presence of reduced sun exposure) up to 1000 IU/day (i.e. in presence of multiple risk factors for vitamin D deficiency).
- In cases of poor compliance, supplementation with intermittent dosing (weekly or monthly doses for a cumulative monthly dose of 18000–30000 IU of vitamin D) can be considered, starting from children aged 5–6 years and particularly during adolescence.
- We suggest vitamin D supplementation from the end of fall to the beginning of spring (November–April) in children and adolescents with reduced sun exposure during summer. We suggest continuous vitamin D supplementation in cases of permanent risk factors for vitamin D deficiency.
- Individuals on anticonvulsants, oral corticosteroids, antimicotics and antiretroviral drugs should receive at least 2–3 times more vitamin D than the daily requirement recommended for age.
- We endorsed as Tolerable Upper Intake Levels of vitamin D those proposed by EFSA in 2012 (1000 IU/day for infants; 2000 IU/day for children ages 1 to 10 years; 4000 IU/day for children and adolescents ages 11 to 17 years).
- We recommend against using vitamin D metabolites and their analogs (calcifediol, alfacalcidol, calcitriol, and dihydrotachysterol) for the routine vitamin D supplementation. The administration of these compounds increases the risk of hypercalcemia and is not able to maintain and/or restore vitamin D stores.
- We recommend against routine 25(OH)D testing in children and adolescents. We suggest to measure serum 25(OH)D levels in presence of multiple risk factors for vitamin D deficiency. Vitamin D status should be monitored at least yearly in subjects that require supplementation during the whole year because affected from pathological conditions or receiving drugs affecting vitamin D metabolism (Table 7).

### Skeletal actions of vitamin D

#### Nutritional rickets

- Children of immigrants living in industrialized countries are at increased risk of nutritional rickets because they usually present several risk factors for vitamin D deficiency such as prolonged breastfeeding without vitamin D supplementation, increased skin pigmentation, reduced sun exposure due to cultural habits (i.e. veiling), and reduced intestinal calcium uptake due to an excessive intake of high-phytates foods.
- In suspicion of nutritional rickets we recommend the assessment of serum 25(OH)D, parathyroid hormone, alkaline phosphatase, calcium, and phosphorus levels and an X-rays evaluation of metaphyseal sites (wrists and ankles) to confirm the diagnosis.
- Treatment of nutritional rickets is based on the administration of vitamin D (2000 IU/day in patients aged less than 1 year, 3000–6000 IU/day in patients aged 1 to 12 years and 6000 IU/day in patients older than 12 years for a minimum of 3 months) and calcium (30–75 mg/kg/day of elemental calcium in 3 divided doses, starting at a higher dose and weaning down to the lower end of the range over 2–4 weeks).
- Vitamin D metabolites and their analogs (calcifediol, alfacalcidol, calcitriol, and dihydrotachysterol) are not recommended for routine treatment of nutritional rickets.
- Daily administration of vitamin D is preferred in infants. Intermittent administration of vitamin D may be considered in children and adolescents with poor compliance with daily treatment (i.e. 50000 IU weekly for 6–8 consecutive weeks or 100000 IU monthly for 3–4 consecutive months).
- We recommend against the administration of a single large dose of vitamin D > 300000 IU.
- After rickets healing, we recommend to continue vitamin D supplementation according to age (400–1000 IU/day in the first year of life and 600–1000 IU/day from 1 to 18 years).

#### Bone health

- Vitamin D directly influences bone mass acquisition contributing to the regulation of calcium-phosphorus metabolism, and indirectly stimulating the development of muscle tissue.
- Available evidence suggests a positive effect of vitamin D supplementation on bone mass acquisition in children and adolescents with vitamin D deficiency.
- Recent studies suggest a relationship between maternal vitamin D status during pregnancy and bone mass of the fetus and the newborn. Uncertain evidence exists on the relationship with bone mass later in life until the acquisition of bone mass.

### Extraskeletal actions of vitamin D

#### Respiratory infections

- Recent studies suggest an association between vitamin D deficiency and the severity or the incidence of respiratory infections in children. However, because current evidence is weak, we suggest against vitamin D administration as a therapy for respiratory infections, beyond the correction of a documented vitamin D deficiency.
- No definitive evidence exists to recommend vitamin D supplementation for prevention of respiratory infections (non-associated with wheezing) or recurrent respiratory infections. However, some studies suggested that serum 25(OH)D higher than those needed for the prevention of nutritional rickets may be necessary to exert a regulatory effect on the immune system.
- We recommend against routine 25(OH)D testing in children with respiratory infections.
- A single RCT suggested that vitamin D supplementation may help in preventing non complicated acute otitis media. However, considering this limited evidence, evaluation of serum 25(OH)D levels may be reasonable before starting supplementation in these children.

#### Other infections

- Reduced serum 25(OH)D levels are found in children with different types of infectious diseases (tuberculosis, HIV, viral hepatitis, acute diarrhea). However, available evidence does not support a causal relationship between hypovitaminosis D and infections.
- We suggest the evaluation of vitamin D status only in patients affected by tuberculosis or HIV infection, as they may benefit from vitamin D supplementation, in particular due to concomitant treatments that influence vitamin D metabolism.
- We recommend against vitamin D supplementation to reduce incidence or severity of non-respiratory infections in children. However, as few studies showed that vitamin D supplementation improves clinical parameters in patients with active tuberculosis or HIV infection, more rigorous and extensive studies are needed to evaluate the role of vitamin D supplementation in infectious diseases, considering also the setting of care, children’s age and nutritional status, potential co-infections and compliance with anti-infective therapy.

#### Asthma

- Recent meta-analyses did not find an association between 25(OH)D levels in cord blood or during pregnancy and the development of asthma later in life.
- Several studies showed a relationship between latitude, prevalence of hypovitaminosis D and increase in allergic asthma prevalence in the pediatric population.
- Low serum 25(OH)D levels are associated with increased asthma severity and increased risk of asthma exacerbations requiring hospitalization or medical treatment. Recent meta-analyses suggest that supplementation with vitamin D (500–2000 IU/day) may reduce the risk of asthma exacerbation.
- We recommend against routine 25(OH)D testing in children with asthma.

#### Atopic dermatitis and allergic diseases

- Low serum 25(OH)D levels are associated with increased incidence and/or severity of AD in children in most but not all studies. Indeed, a pathogenetic role of vitamin D in AD has yet to be demonstrated.
- We recommend against routine 25(OH)D testing in children with AD. Serum 25(OH)D evaluation may be considered in children with severe AD unresponsive to common therapy and multiple risk factors for vitamin D deficiency.
- In pediatric population there is limited evidence regarding a possible favourable effect of vitamin D supplementation on several aspects of AD.
- A short trial of vitamin D supplementation can be considered in patients with severe AD unresponsive to common therapy, especially in the late winter-early spring period. In presence of documented vitamin D deficiency, we recommended adequate treatment to restore vitamin D status, followed by supplementation at doses recommended for age.
- Vitamin D supplementation for primary prevention of allergic diseases, including food allergies, remains an attractive area of study, but actual evidence does not allow to make any recommendation.

#### Type 1 diabetes mellitus

- Children and adolescents with T1DM are at risk for vitamin D deficiency.
- Recommended vitamin D intakes in patients with T1DM are the same as those for the healthy pediatric population.
- Recommendations for vitamin D treatment in subjects with T1DM and vitamin D deficiency are the same as those for the healthy pediatric population.
- At present, there is no evidence that vitamin D supplementation may delay the development of T1DM or may ameliorate its clinical features. However, we recommend to maintain vitamin D intake for age and to treat vitamin D deficiency if found.
- We recommend against routine 25(OH)D testing in children with T1DM.

#### Inflammatory bowel diseases

- In patients with IBDs (Crohn’s disease or ulcerative colitis) we suggest to evaluate serum 25(OH)D levels at diagnosis and at least yearly, preferably in the late winter-early spring period.
- We recommend continuous vitamin D supplementation at higher doses than those recommended for age (at least 1000–1500 IU/day).
- We recommend to treat vitamin D deficiency with daily vitamin D at higher doses than those recommended for otherwise healthy pediatric population (at least 2000–4000 IU/day), for a minimum of 6–8 weeks.
- Intermittent bolus doses of vitamin D for a cumulative dose of at least 400000 IU should be reserved for IBDs patients with vitamin D deficiency and poor compliance with daily treatment.
- After achieving vitamin D sufficiency, we recommend to continue with vitamin D supplementation at higher doses than those recommended for age (at least 1000–1500 IU/day).

#### Celiac disease

- We recommend to evaluate serum 25(OH)D levels in patients with CD at diagnosis and 6–12 months after the beginning of gluten-free diet if deficiency has been found. We suggest against further 25(OH)D evaluations in case of sufficient 25(OH)D levels or documented normalization of vitamin D status and strict adherence to the gluten-free diet.
- Recommendations for treatment of vitamin D deficiency in patients with newly diagnosed CD are equivalent to those for IBDs patients, as both these conditions are associated with intestinal malabsorption.
- As gluten-free diet restores normal intestinal absorption, after treatment of deficiency we recommend vitamin D supplementation according to the modalities and requirements for otherwise healthy children and adolescents.

#### Obesity and metabolic syndrome

- Epidemiological studies showed that obese children and adolescents are at high risk for vitamin D deficiency.
- Obesity is the cause and not the effect of this association because of the deposition of vitamin D in adipose tissue with consequent reduction in serum 25(OH)D levels.
- It is uncertain if vitamin D deficiency may worsen metabolic profile of obese children and adolescents.
- We suggest against vitamin D administration to improve obesity-related complications, given inconsistent results and the small number of studies.
- We suggest vitamin D supplementation at higher doses than those recommended for age (1000–1500 IU/day) from the end of fall to the beginning of spring (November–April) in obese children and adolescents to ensure an adequate vitamin D status. Obese subjects with reduced sun exposure during summer should receive vitamin D supplementation throughout the year. Finally, sensible sunlight exposure and outdoor physical exercise should be encouraged in obese children and adolescents.
- We recommend against routine evaluation of 25(OH)D levels in obese subjects. If an obese individual does not receive vitamin D supplementation and has a sedentary indoor lifestyle with consequent reduced sun exposure, serum 25(OH)D evaluation may be considered to confirm vitamin D deficiency and start adequate treatment.
- In obese subjects with vitamin D deficiency we recommend vitamin D treatment at higher doses than those recommended for otherwise healthy pediatric population (at least 2000–4000 IU/day), for a minimum of 6–8 weeks.

#### Autism

- Autistic individuals frequently develop nutritional deficiencies, especially in presence of food selectivity; causal relationship with vitamin D deficiency is uncertain.
- We recommend against routine evaluation of 25(OH)D levels in children with autism.
- We recommend against vitamin D treatment to improve patient’s performance considering the limited and conflicting available evidence.

#### Depression

- Epidemiological studies that evaluated an association between vitamin D deficiency and depression are lacking at present.
- We recommend against routine evaluation of 25(OH)D levels in children with depression.
- We recommend against vitamin D administration to improve mood.

### Pregnancy and breastfeeding

- We suggest against routine screening of serum 25(OH)D levels in all pregnant and breastfeeding women. We suggest to consider serum 25(OH)D testing in women with multiple risk factors for vitamin D deficiency, particularly if not receiving vitamin D supplementation, and/or with specific conditions possibly affecting pregnancy course (Table 9).
- We recommend vitamin D supplementation in all pregnant and breastfeeding women at a dose of 600 IU/day. Women with risk factors for vitamin D deficiency should receive higher dosages (1000–2000 IU/day).
- We suggest to start vitamin D supplementation at the beginning of pregnancy and continue for the entire duration of pregnancy and lactation.

### Appendix. Management of asymptomatic vitamin D deficiency and insufficiency

- In children and adolescents with asymptomatic vitamin D deficiency [25(OH)D <  20 ng/ml] we recommend the administration of 2000 IU/day or 50000 IU/week of vitamin D_2_ or D_3_ for 6–8 weeks (8 weeks in adolescents) in order to obtain a sufficient vitamin D status [25(OH)D ≥ 30 ng/ml].
- After completion of treatment, serum 25(OH)D concentrations should be revaluated.
- In presence of adequate vitamin D status [25(OH)D ≥ 30 ng/ml], we recommend to continue vitamin D supplementation as recommended for age.
- In subjects with asymptomatic vitamin D deficiency assuming drugs interfering with vitamin D metabolism (anticonvulsants, oral corticosteroids, antifungals like ketoconazole, anti-retroviral drugs) we recommend vitamin D treatment at higher doses than those recommended for otherwise healthy pediatric population (at least 2000–4000 IU/day), for a minimum of 6–8 weeks.
- Considering available evidence, particularly the lack of Italian studies, at present we do not recommend other modalities different from daily or weekly administration of vitamin D to treat asymptomatic vitamin D deficiency.
- In case of detection of asymptomatic vitamin D insufficiency [25(OH)D between 20 and 29 ng/ml], particularly in subjects at risk for vitamin D deficiency, we recommended to start vitamin D supplementation according to the modalities and requirements recommended for age.

## Abbreviations

1,25(OH)_2_D: 1,25-dihydroxyvitamin D
25(OH)D: 25-hydroxyvitamin D
AAP: American Academy of Pediatrics
AD: Atopic dermatitis
BMC: Bone mineral content
BMD: Bone mineral density
CD: Celiac disease
DXA: Dual-energy X-ray absorptiometry
EFSA: European Food Safety Authority
ESPGHAN: European Society for Paediatric Gastroenterology Hepatology and Nutrition
FEV1: Forced expiratory volume in the 1st second
HIV: Human immunodeficiency virus
IBD: Inflammatory bowel disease
IL: Interleukin
LC-MS/MS: Liquid chromatography-tandem mass spectrometry
PBM: Peak bone mass
pQCT: Peripheral quantitative computed tomography
PTH: Parathormone
RCT: Randomized controlled trial
SACN: Scientific Advisory Committee on Nutrition
T1DM: Type 1 diabetes mellitus
VDR: Vitamin D receptor
VLBW: Very low birth weight
